# Pan-cancer analysis reveals ELFN1 as a novel prognostic biomarker and immunotherapeutic target associated with tumor microenvironment remodeling and promoting malignant phenotypes in colorectal cancer

**DOI:** 10.3389/fonc.2025.1583277

**Published:** 2025-11-20

**Authors:** Sha-Sha Hu, Tian-Yuan Tan, Wei Yan, Yu Wu, Xin-Nian Li, Fu-Jin Liu, Bo Wang

**Affiliations:** 1 Department of Pathology, Hainan Affiliated Hospital of Hainan Medical University, (Hainan General Hospital), Haikou, China; 2 Department of Pathology, School of Basic Medical Sciences, Southern Medical University, Guangzhou, China; 3 Medical Laboratory Center, Hainan Affiliated Hospital of Hainan Medical University, (Hainan General Hospital), Haikou, China

**Keywords:** ELFN1, pan-cancer analysis, tumor microenvironment, immunotherapy, molecular docking, colorectal cancer

## Abstract

**Background:**

Extracellular leucine rich repeat and fibronectin type III domain containing 1 (ELFN1), a transmembrane protein implicated in tumorigenesis and therapy resistance, remains mechanistically undefined as a pan-cancer target. In this study, we aimed to elucidate the function and potential mechanism of action of ELFN1 across cancers.

**Methods:**

Through integrative analysis of TCGA and GTEx datasets, we systematically characterized ELFN1 across 33 cancer types, including its expression patterns, prognostic value, mutation landscape, methylation modifications, protein-protein interaction (PPI) networks, and the relationship between ELFN1 expression and immune infiltration. KEGG enrichment analysis was also performed to predict the functions and associated cellular pathways of ELFN1. In addition, the molecular docking tool was used to analyze the affinities between ELFN1 protein and drugs. Finally, we assessed the effect of ELFN1 knockdown on colorectal cancer (CRC) cells using *in vitro* experiments.

**Results:**

Our study revealed significant dysregulation of ELFN1 across various cancer types, with notable diagnostic and prognostic utility in most cancers analyzed. Mechanistically, ELFN1 expression was associated with DNA methylation, DNA repair, genomic instability, and tumor microenvironment (TME) scores in multiple cancer types. Furthermore, Drug sensitivity profiling linked ELFN1 to ABT-737 susceptibility and benzaldehyde resistance through molecular docking. In CRC cells, ELFN1 knockdown significantly inhibited tumor proliferation, migration, and motility.

**Conclusion:**

The expression level of ELFN1 may provide insights into tumor development and progression in multiple cancers, including CRC, highlighting its potential utility as an effective prognostic biomarkers and immunotherapeutic targets.

## 1 Introduction

Cancer remains a significant global public health issue and has become one of the leading causes of death, posing a serious threat to human health (1). The development of malignant tumors is primarily driven by a combination of genetic and environmental factors, alongside a highly complex tumor microenvironment (TME), which makes these tumors particularly challenging to eliminate (1–3). Traditional cancer treatments, including surgery, radiotherapy, and chemotherapy, are the cornerstone of clinical practice (4, 5). However, their overall efficacy has not yet met expectations, leading to the emergence of immunotherapy as a promising alternative for cancer management (6, 7). Despite its potential, current immunotherapies exhibit low response rates, necessitating the identification of reliable predictive biomarkers to optimize treatment outcomes. The heterogeneous nature of tumors and their microenvironments contributes substantially to the limited efficacy of immunotherapy. In breast cancer, particularly inflammatory breast cancer (IBC), the complex immune landscape frequently exhibits resistance to immune checkpoint blockade (ICB) therapies. This resistance stems from multiple mechanisms, including tumor-specific genomic alterations and the loss of tumor-specific antigens that enable immune evasion and sustained proliferation despite therapeutic intervention (8, 9). Similar challenges emerge in other malignancies; bladder urothelial carcinoma, for example, demonstrates reduced responsiveness to immune checkpoint inhibitors despite high immune cell infiltration, indicating that mere immune cell presence fails to guarantee therapeutic success (10). Pseudoprogression further complicates treatment evaluation, as tumors may transiently enlarge before responding, potentially leading to premature discontinuation of effective therapy. These observations necessitate revised clinical response criteria that accommodate immunotherapy’s distinct temporal patterns (11). Additionally, the restricted subset of responders observed across melanoma and other cancers highlights the need for combinatorial strategies integrating immunotherapy with complementary treatment modalities (12). Given the intricate and diverse mechanisms underlying cancer progression, comprehensive analyses of genetic and biological characteristics are essential for improving clinical treatment strategies and prognostic predictions.

*Extracellular leucine-rich repeat and fibronectin type III domain-containing 1 (ELFN1)* is a gene that encodes a protein with several structural features in its extracellular region, including multiple leucine-rich repeats (LRRs) and a fibronectin type III (FN3) domain (13). Previous studies have demonstrated that *ELFN1* plays a role in immune regulation and various biological processes in malignant tumors, including melanoma, colorectal, breast, and ovarian cancers (14–17). In silico analysis of pan-cancer single-cell RNA sequencing datasets has revealed that *ELFN1* is predominantly expressed in cancer-associated fibroblasts (CAFs) and endothelial cells across multiple cancer types, such as breast, lung, colorectal, and ovarian cancers (14, 18).

Immune checkpoint blockade (ICB) therapies have revolutionized the treatment landscape for advanced cancers (19). Extensive research has been directed toward incorporating immune-related factors into predictive models to evaluate the efficacy of ICB therapies, either alone or in combination (20). However, the limited ability of ICB therapies to induce durable clinical responses underscores the immunosuppressive complexity of the TME (21). The TME plays a pivotal role in tumor initiation, progression, and inhibition (22). It encompasses a highly intricate ecosystem surrounding tumor cells, comprising diverse stromal and immune cell populations (23). Among these, CAFs are particularly abundant and interact dynamically with tumor cells and the surrounding TME, contributing to tumor growth, metastasis, drug resistance, TME remodeling, and immunosuppression (24). As a result, CAFs are increasingly regarded as promising therapeutic targets to enhance the efficacy of immunotherapies.

Although the current evidence supports the hypothesis that *ELFN1* may influence various cancer types, systematic pan-cancer analyses exploring its role are still lacking. This study aimed to address this gap by conducting a multi-omics pan-cancer investigation of *ELFN1* using a comprehensive array of datasets and tools to examine its relationship with clinical features and multi-omics heterogeneity. The analysis focused on aspects such as abnormal expression patterns, prognostic significance, clinical correlations, tumor mutation burden (TMB) and microsatellite instability (MSI) associations, TME characteristics, tumor-immune interactions, genetic alterations, DNA methylation, biological functions, drug susceptibility, and molecular docking. Additionally, the functional role of *ELFN1* in the malignant phenotype of CRC was validated through *in vitro* experiments. This study seeks to uncover the prognostic and immunological roles of *ELFN1* in cancer, providing insights into its potential as a biomarker and immunotherapeutic target.

## 2 Materials and methods

### 2.1 Study on ELFN1 expression and subcellular localization

The Human Protein Atlas (HPA) database (https://www.proteinatlas.org, accessed on November 7, 2024) and UALCAN (https://ualcan.path.uab.edu, accessed on November 7, 2024) were used to investigate the RNA expression levels of *ELFN1* in normal human tissues, as well as its RNA and protein expression levels in normal and tumor cell lines. Additionally, the subcellular localization and single-cell expression of *ELFN1* in tumor cell lines were analyzed. The TIMER2.0 database (http://timer.cistrome.org, accessed on November 7, 2024) and Sangerbox3.0 database (http://sangerbox.com/home.html, accessed on November 7, 2024) were used to obtain expression levels of the *ELFN1* gene in various cancer tissues. *ELFN1* expression data in normal and tumor samples were derived from the TCGA (http://cancergenome.nih.gov) and GTEx (http://commonfund.nih.gov/GTEx/) databases. The ELFN1 expression data were used to construct ROC curves, with the area under the curve (AUC) serving as an indicator of its accuracy. For details regarding the naming and abbreviations of the 33 tumor types included in this study, see **Supplementary Table S1**.

### 2.2 Prognostic and clinical correlation analysis of ELFN1

Pan-cancer datasets standardized to a unified format were downloaded from the UCSC database (https://xenabrowser.net/), providing *ELFN1* expression data across various cancers along with OS, PFS, DSS, DFI, and PFI data for corresponding samples. The Cox proportional hazards regression model was established using the coxph function in the R’package survival (version 3.2-7) to analyze the relationship between ELFN1 expression and prognosis in each cancer type. Statistical significance of prognosis was assessed using the log-rank test. Kaplan-Meier curves were obtained using the GEPIA2.0 database, TIMER2.0 database, Sangerbox3.0 database, Genomic Cancer Analysis database (GSCA) (25) (https://guolab.wchscu.cn/GSCA, accessed on November 12, 2024), and KM-plot database (https://kmplot.com/analysis/, accessed on November 12, 2024) to evaluate prognosis. The GSCA database was also used to analyze the relationship between *ELFN1* expression and clinical pathological staging and classification.

### 2.3 Genomic alterations and mutation burden analysis

The “Mutation” module of the GSCA database was utilized to examine the association between ELFN1 and CNVs or SNVs in different cancer types, as well as to assess the relationship between ELFN1 CNVs and prognosis in various cancers. The R’package maftools was employed to evaluate TMB. Data related to aneuploidy, neoantigens, HRD, and MSI were acquired from previous studies (26)and used to analyze the correlation between these features and ELFN1 expression.

### 2.4 DNA mismatch repair, stemness, and epigenetic modification analysis

The relationship between *ELFN1* and the expression of five MMR (27)genes and three DNMTs (28) was visualized. The ARIEL3 (29) clinical trial was referenced to retrieve 30 HRR-related genes, and the correlation between these genes and *ELFN1* mRNA levels was evaluated using the GEPIA2.0 tool. Tumor stemness scores, calculated based on methylation characteristics of individual tumors and obtained from previous studies (30), were integrated with stemness indices and gene expression data to assess the association between these values and *ELFN1* mRNA expression. Heatmaps were used to evaluate the correlation between *ELFN1* and the expression of 44 genes involved in N1-methyladenosine (m1A), 5-methylcytosine (m5C), and N6-methyladenosine (m6A) modifications (31).

### 2.5 ELFN1 DNA methylation analysis

The “Mutation” module of the GSCA database was used to assess the relationship between methylation and *ELFN1* mRNA expression levels, as well as the correlation between *ELFN1* methylation and OS, PFS, DSS, and DFI in patients. The TIDE methylation module was employed to evaluate the relationship between *ELFN1* promoter methylation and CTLs.

### 2.6 Pan-cancer analysis of ELFN1’s immunological role

The ESTIMATE algorithm was used to calculate immune, stromal, and ESTIMATE scores for 33 cancer types (32). The correlation between *ELFN1* and previously identified immune checkpoint markers was examined at the mRNA level (26, 33). The association between *ELFN1* and five categories of immune pathways [MHC molecules (21), chemokine receptors (18), chemokines (41), immunosuppressive genes (24), and immunostimulatory genes (46)] was analyzed across various cancer types. Using the TIMER2.0 database, the correlation between *ELFN1* mRNA and the expression of 21 immune cell types was determined.

### 2.7 Single-cell sequencing analysis of ELFN1

The TISCH database (http://tisch.comp-genomics.org/, accessed on December 1, 2024) was used to automatically parse and manage tumor single-cell RNA-seq datasets from the GEO database. The CancerSEA database (34) (http://biocc.hrbmu.edu.cn/CancerSEA/, accessed on December 1, 2024) was utilized to explore the mean correlation between *ELFN1* and 14 functional states across 93,475 cancer single cells from 27 human cancer types. A correlation strength threshold of 0.3 was applied.

### 2.8 PPI network and enrichment analysis of ELFN1-related genes

The PPI network of ELFN1-binding proteins was obtained from the STRING database (https://cn.string-db.org/, accessed on December 1, 2024). The “Similar Gene Detection” module of the GEPIA2.0 database was used to generate the top 100 *ELFN1*-related target genes in TCGA tumors. GO and KEGG analyses of *ELFN1*-related target genes were then performed and visualized using the SangerBox3.0 platform.

### 2.9 Drug sensitivity and molecular docking analysis

Drug sensitivity analysis was based on the relationship between gene expression and IC50 of drugs. Processed datasets, including RNA expression and drug activity data from NCI-60 cancer cell lines, were obtained from the CellMiner (35) (https://discover.nci.nih.gov/cellminer/, accessed on December 17, 2024). FDA-approved drugs or those undergoing clinical trials were extracted for further analysis. Positive correlations indicated that higher gene expression might lead to drug resistance, while negative correlations suggested drug sensitivity. GSCA database, which integrates mRNA expression data and drug sensitivity data from GDSC database and CTRP database, was used to perform a drug sensitivity analysis of *ELFN1* in pan-cancer (25).

To analyze the interaction patterns and binding affinities between proteins and potential drugs, the protein structure predicted by AlphaFold3 was used as a template. Semi-flexible docking was performed using the Autodock Vina software. Among the ten generated conformations, the one with the lowest binding free energy was selected, and the results were visualized using Pymol software. ELFN1 protein FASTA sequences were obtained from the NCBI protein database (**Supplementary Material S1**).

### 2.10 Experimental methods

#### 2.10.1 Cells culture

The human CRC cell lines HCT8, Caco-2, M5, HCT116, LoVo, SW480 and DLD1, and NCM460, a normal colorectal cell, were obtained from the American Type Culture Collection (ATCC). All cell lines were cultured in RPMI1640 medium (Invitrogen, Carlsbad, CA, USA) containing 10% fetal bovine serum (FBS, Invitrogen) at 37°C under 5% CO_2_.

#### 2.10.2 RNA extraction and real-time PCR

Total RNA was extracted from tissue samples and cell lines using Trizol reagent (TaKaRa, Dalian, China) according to the manufacturer’s instructions. cDNA was synthetized using the Prime-Script RT Reagent Kit (TaKaRa). Real-time PCR was performed using SYBR Premix Ex Taq II (TaKaRa) and measured in the ABI PRISM 7500 Sequence Detection System (Applied Biosystems, CA, USA). The assay was performed in triplicate for each case to allow the assessment of technical variability. GAPDH was used as an internal control. The primers sequences are: ELFN1 primers 5’-TGGCAACCTCACGTACCTCA-3’ (sense) and 5’- CAGGTCGATGTTGACGATGTT-3’ (antisense); GAPDH primers 5’- GGAGCGAGATCCCTCCAAAAT-3’ (sense) and 5’- GGCTGTTGTCATACTTCTCATGG’ (antisense).

#### 2.10.3 Cell immunofluorescence

Cells were cultured in glass-bottom confocal dishes until reaching 50-70% confluence. Cell lines were fixed with 4% paraformaldehyde (PFA) and permeabilized in phosphate-buffered saline (PBS) containing 0.5% Triton X-100. Blocking was performed using 5% bovine serum albumin (BSA). Subsequently, samples were incubated with primary antibodies against ELFN1 (Invitrogen, catalog#PA5-84721, 1:100) at 4°C overnight, and subsequently incubated with corresponding Alexa Fluor-conjugated secondary antibodies. After 1 h, cell nuclei were stained with 4, 6-diamidino-2-phenylindole (DAPI; 5 μg/mL). Microscopic images of cells were obtained using a Leica inverted fluorescence microscope.

#### 2.10.4 Stable transfection and knockdown

To achieve gene knockdown, short hairpin RNA (shRNA) targeting ELFN1 was delivered into cells using lentiviral vectors. Lentiviral particles were generated by co-transfecting HEK293T cells with the shRNA-expressing plasmid, packaging plasmid (psPAX2), and envelope plasmid (pMD2.G) using Lipofectamine 3000 (ThermoFisher Scientific). After 24–48 hours of transfection, the viral supernatant was collected, filtered, and stored at -80°C. Target cells were infected with the lentivirus in the presence of Polybrene to enhance transduction efficiency. After incubation for 24–48 hours, the medium was replaced, and puromycin was added to select for successfully transduced cells. The knockdown efficiency of ELFN1 was confirmed using quantitative reverse transcription PCR (qRT-PCR). The shRNA sequence targeting ELFN1 was: CCGGTGTTCACGCTCACCAACTACACTCGAGTGTAGTTGGTGAGCGTGAACATTTTTG.

#### 2.10.5 Cell proliferation and colony formation assays

For the cell proliferation assay, cells were seeded into 96-well plates at a density of 1×10³ cells per well. After 24 hours, cell proliferation was evaluated using the CCK-8 assay (Dojindo Laboratories, Kyushu Island, Japan) according to the manufacturer’s instructions.

For the colony formation assay, cells were plated in 6-well plates at a density of 1×10³ cells per well and cultured in medium containing 10% FBS for 2 weeks. Colonies were fixed with methanol, stained with Giemsa, and counted under a microscope.

#### 2.10.6 Wound-healing and invasion assays

For the wound-healing assay, a scratch was created in a confluent cell monolayer using a 10 μL pipette tip, and the wound closure was observed at 0 and 48 hours. Migration was quantified by measuring the distance covered by cells migrating into the wound area.

Cell invasion potential was assessed using a transwell chamber system with Matrigel-coated membranes. Cells were suspended in serum-free medium at a standardized concentration of 1×10^5^ cells/mL and carefully seeded into the upper chambers. The lower chamber was filled with complete culture medium supplemented with 10% fetal bovine serum, serving as a chemoattractant gradient. Following incubation at 37 °C for 48 hours, non-invasive cells remaining on the upper membrane were removed by gentle wiping. Cells that successfully migrated and invaded through the Matrigel matrix were fixed with 10% neutral buffered formalin and subsequently stained with 0.1% crystal violet solution for 30 minutes. Invasive cell quantification was performed by counting the stained cells under a standard light microscope, with multiple random fields analyzed to ensure statistical reliability.

### 2.11 Statistical analysis

Statistical analyses included two-tailed Student’s *t*-*test* and one-way ANOVA. Kaplan-Meier curves and log-rank tests or Cox proportional hazard regression models were employed when conducting survival analyses. Pearson or Spearman correlation coefficient values were used to evaluate relationships between variables, with |r| = 0.3 being considered indicative of a relevant correlative relationship. *P* < 0.05 was defined as statistically significant.

## 3 Results

### 3.1 Dysregulated expression of ELFN1 in various tumor tissues

**Figure 1:**
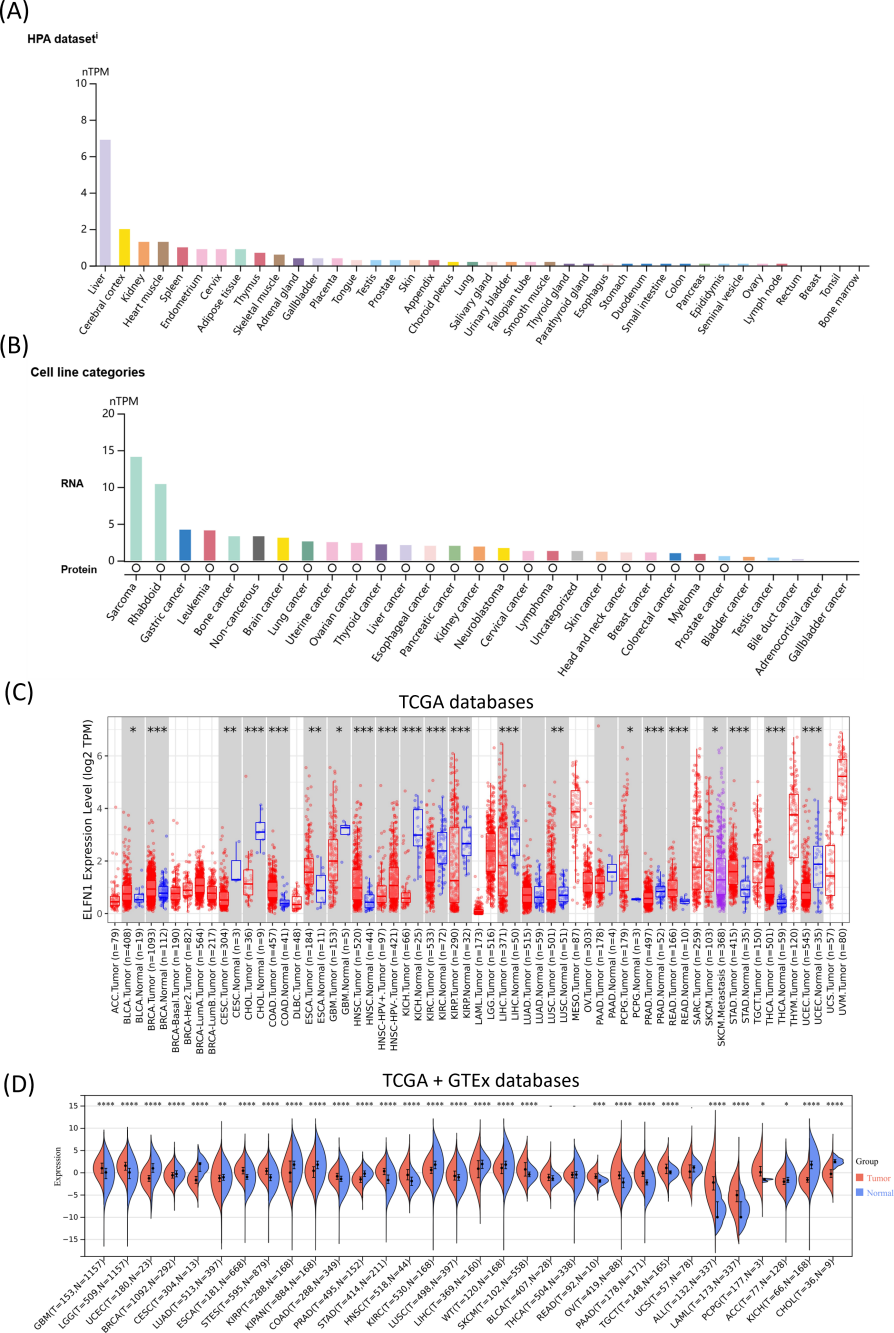
*ELFN1* expression levels in normal tissues and cancers. **(A)***ELFN1* mRNA expression profiles in normal human tissues from the HPA database. **(B)***ELFN1* mRNA and protein expression in cancer cell lines from the HPA database. **(C)***ELFN1* mRNA expression levels across 33 tumor types from the TCGA database via the TIMER2.0 portal. **(D)***ELFN1* mRNA expression levels in different tumors and corresponding normal tissues from the TCGA and GTEx databases using the SangerBox portal. *P < 0.05, **P < 0.01, ***P < 0.001, ****P < 0.0001.

We first evaluated the RNA expression levels of *ELFN1* in normal human tissues and found that it is expressed in most tissues. Notably, *ELFN1* showed high expression in normal liver tissue, while its expression was relatively low in other normal tissues (**Figure 1A**). At both the RNA and protein levels, ELFN1 expression was detected in various tumor cell lines, with relatively high levels observed in sarcoma, rhabdoid (RB), gastric cancer, lymphoma, and bone cancer cell lines (**Figure 1B**). Immunofluorescence analysis further confirmed that ELFN1 is localized in both the nucleus and cytoplasm of MCF7 and U2OS cells (**Supplementary Figure S1A**). We next compared *ELFN1* expression between tumor tissues and their corresponding normal tissues using the TCGA and GTEx databases. The results (**Figures 1C, D**) showed that *ELFN1* was significantly upregulated in 18 tumor types, including acute lymphoblastic leukemia (ALL), bladder urothelial carcinoma (BLCA), colon adenocarcinoma (COAD), esophageal carcinoma (ESCA), glioblastoma multiforme (GBM), head and neck squamous cell carcinoma (HNSC), acute myeloid leukemia (LAML), low-grade glioma (LGG), lung squamous cell carcinoma (LUSC), pancreatic adenocarcinoma (PAAD), pheochromocytoma and paraganglioma (PCPG), rectum adenocarcinoma (READ), skin cutaneous melanoma (SKCM), stomach adenocarcinoma (STAD), stomach and esophageal carcinoma (STES), testicular germ cell tumors (TGCT), thyroid carcinoma (THCA), and ovarian serous cystadenocarcinoma (OV). Conversely, *ELFN1* was significantly downregulated in 13 tumor types, including adrenocortical carcinoma (ACC), breast carcinoma (BRCA), cervical squamous cell carcinoma (CESC), cholangiocarcinoma (CHOL), kidney chromophobe (KICH), kidney renal clear cell carcinoma (KIRC), kidney renal papillary cell carcinoma (KIRP), pan-kidney cohort (KIPAN), liver hepatocellular carcinoma (LIHC), lung adenocarcinoma (LUAD), prostate adenocarcinoma (PRAD), uterine corpus endometrial carcinoma (UCEC), and Wilms tumor (WT). Additionally, data from the UALCAN database revealed that ELFN1 protein expression was significantly downregulated in LIHC and GBM compared to normal tissues (**Supplementary Figure S1C**). Single-cell analysis showed that *ELFN1* RNA was predominantly expressed in inhibitory neurons, oligodendrocyte precursor cells, bipolar cells, astrocytes, microglial cells, horizontal cells, and fibroblasts (**Supplementary Figure S1D**). These findings suggest that *ELFN1* is dysregulated in various cancers, highlighting its potential role in cancer progression.

### 3.2 Diagnostic value of ELFN1 in clinical settings

**Figure 2:**
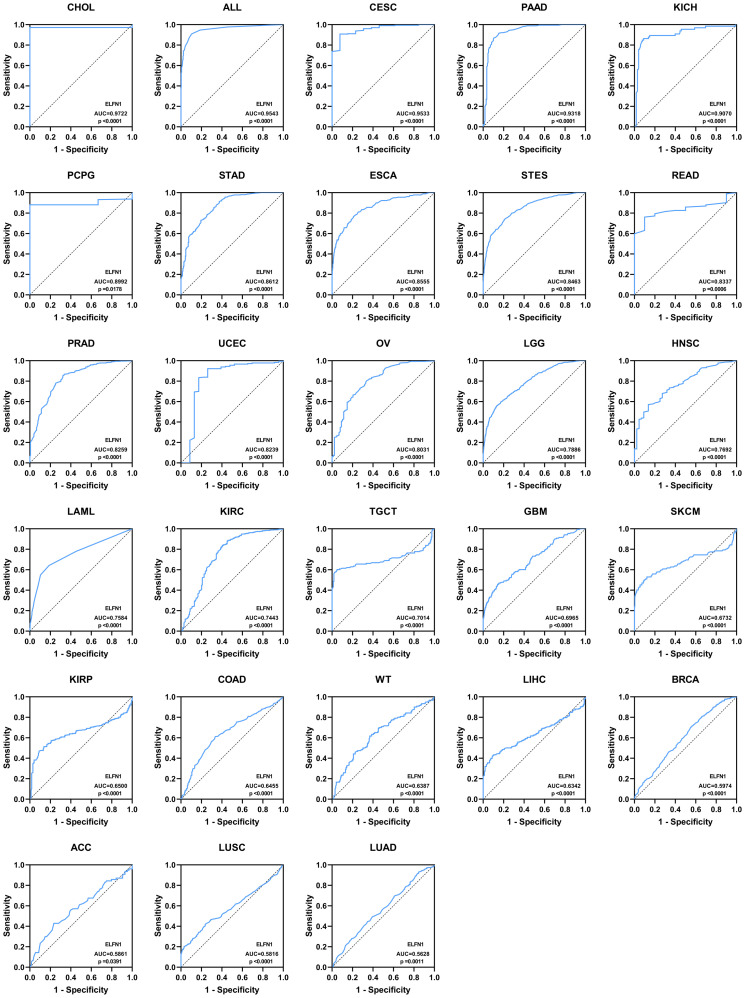
Pan-cancer diagnostic ROC curves.

We analyzed *ELFN1* expression across 33 tumor types at different clinical stages and subtypes. ELFN1 expression was significantly upregulated in the early stages of COAD, LUAD, READ, SKCM, STAD, TGCT, UCS, and UVM (**Supplementary Figure S2A**), while it was significantly downregulated in the early stages of KIRC, LIHC, and THCA (**Supplementary Figure S2A**). These findings suggest that *ELFN1* may have diagnostic value in the early detection of these cancers. Further analysis revealed that *ELFN1* upregulation was closely associated with distant metastasis and lymph node metastasis in COADREAD (**Supplementary Figure S1B**). Significant differences in *ELFN1* expression were also observed among molecular subtypes of BRCA, GBM, HNSC, KIRC, LUAD, and STAD, indicating its potential as a marker for subtype classification (**Supplementary Figure S2B**). ROC curve analysis was performed to evaluate the diagnostic performance of *ELFN1*. Based on AUC thresholds, diagnostic accuracy was classified as high (AUC: 0.9–1.0), moderate (AUC: 0.7–0.9), or low (AUC: 0.5–0.7). As shown in **Figure 2**, ELFN1 demonstrated high diagnostic accuracy in 5 cancers, moderate accuracy in 13 cancers, and low accuracy in 10 cancers.

### 3.3 Prognostic significance of ELFN1 in cancer

**Figure 3:**
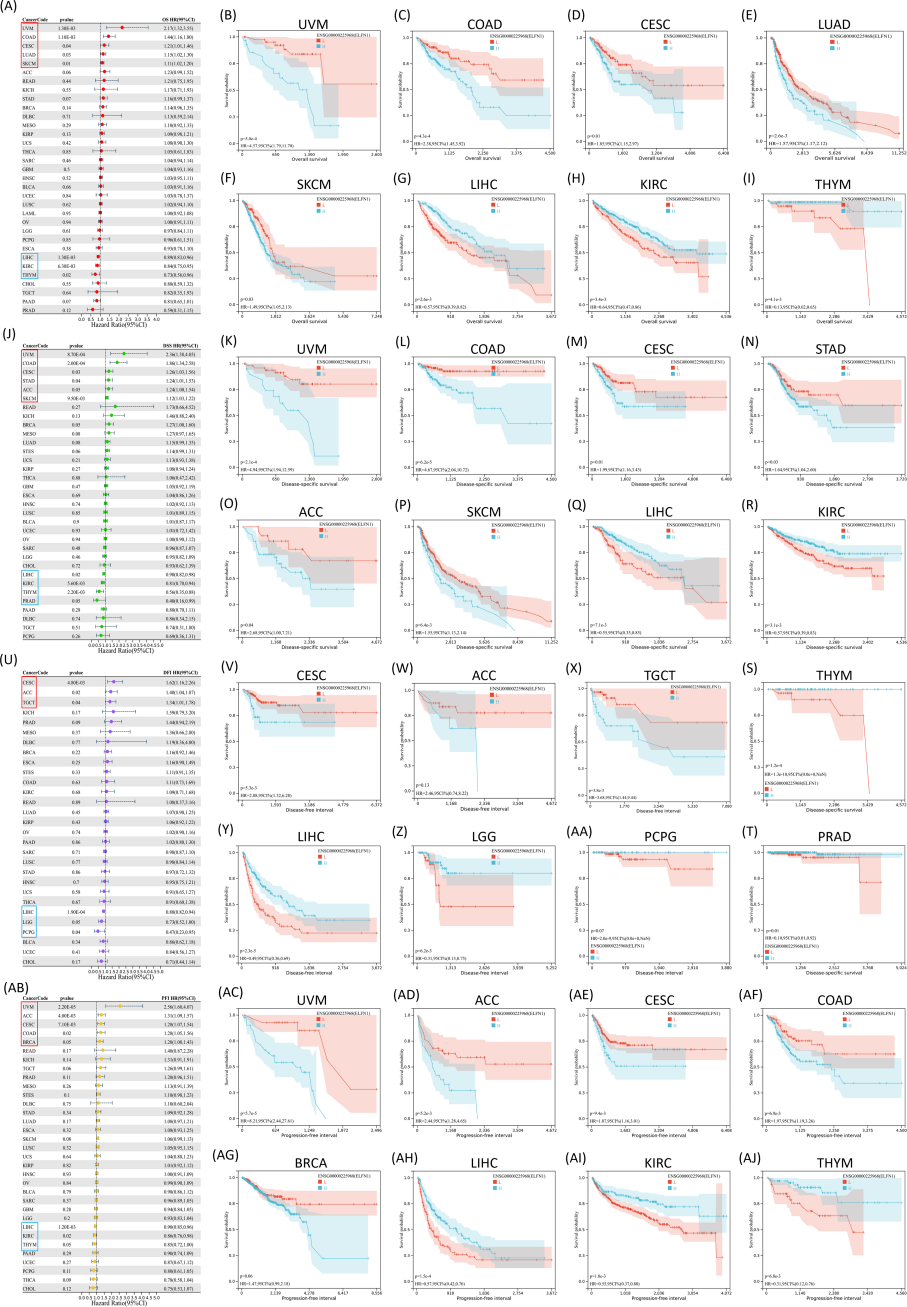
Pan-cancer analyses of *ELFN1* expression and prognostic relevance. **(A–I)** Forest plot **(A)** and Kaplan–Meier curves **(B–I)** showing the association of ELFN1 expression with OS across cancers. **(J–T)** Forest plot **(J)** and Kaplan–Meier curves **(K–T)** for DSS. **(U-AA)** Forest plot **(U)** and Kaplan–Meier curves **(V-AA)** for DFI. **(AB-AJ)** Forest plot **(AB)** and Kaplan–Meier curves **(AC-AJ)** for PFI.

Given its diagnostic potential, we next evaluated the prognostic relevance of *ELFN1* in predicting overall survival (OS), disease-specific survival (DSS), disease-free interval (DFI), and progression-free interval (PFI) across 33 tumor types using the TCGA database. Univariate cox regression analysis revealed that *ELFN1* was significantly associated with poor OS in UVM, COAD, CESC, LUAD, and SKCM, while acting as a protective factor in LIHC, KIRC, and THYM (**Figure 3A**). Similarly, *ELFN1* was a risk factor for poor DSS in UVM, COAD, CESC, STAD, ACC, and SKCM, but served as a protective factor in LIHC, KIRC, THYM, and PRAD (**Figure 3J**). For DFI, *ELFN1* was a risk factor in CESC, ACC, and TGCT, but had protective roles in LIHC and LGG (**Figure 3U**). Regarding PFI, *ELFN1* was a risk factor in UVM, ACC, CESC, COAD, and BRCA, while serving as a protective factor in LIHC, KIRC, and THYM (**Figures 3AB**). Kaplan-Meier survival curves further supported these findings (**Figures 3B-I, K-T, V-AA, AC-AJ**, and **Supplementary Figure S3**). Overall, *ELFN1* overexpression was generally associated with poor prognosis in UVM, COAD, CESC, STAD, ACC, and SKCM patients. Overall, the results demonstrated that the *ELFN1* expression levels were associated with the prognosis of multiple cancers. Moreover, higher *ELFN1* expression was associated with poorer prognosis of CRC patients.

### 3.4 Pan-cancer epigenetic variations of ELFN1

**Figure 4:**
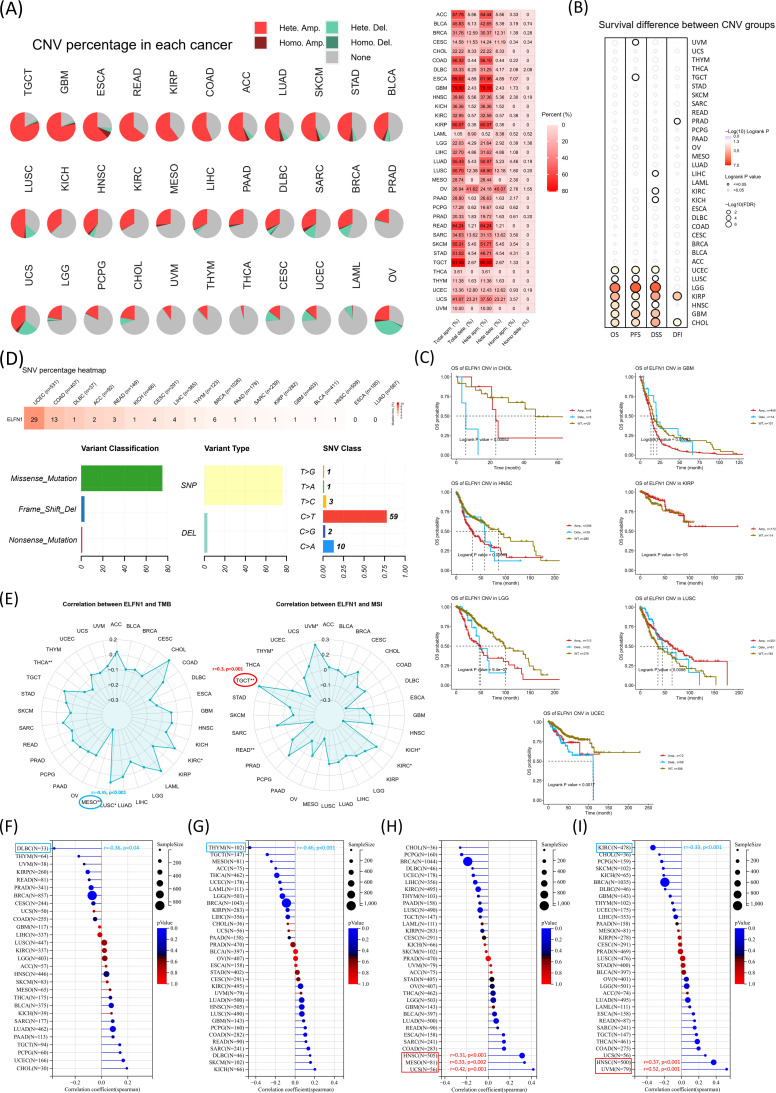
*ELFN1* expression is correlated with genomic instability. **(A)** CNVs of *ELFN1* in pan-cancer. **(B, C)** The relationship between CNVs of *ELFN1* and prognosis in pan-cancer. **(D)** The SNV summary of *ELFN1* in pan-cancer. **(E)** Radar charts representing pan-cancer analyses of the link between *ELFN1* and both TMB (left) and MSI (right). **(F–I)** Lollipop charts were used to visualize correlations between *ELFN1* levels and neoantigen load **(F)**, ploidy **(G)**, HRD **(H)**, and LOH **(I)**.

Genomic strategies provide powerful tools for analyzing cancer (36). To explore genomic alterations of *ELFN1*, we performed pan-cancer analyses of copy number variation (CNV) and single nucleotide variation (SNV) using the TCGA dataset and GSCA portal. *ELFN1* exhibited a high CNV rate across cancers. Amplifications were primarily observed in TGCT, GBM, ESCA, READ, KIRP, COAD, ACC, LUAD, SKCM, STAD, BLCA, and LUSC, while deep deletions were common in OV and UCS (**Figure 4A**). High SNV rates were detected in UCEC and COAD, with missense mutations and C>T substitutions being the most frequent (**Figure 4D**). Survival analysis revealed that high *ELFN1* CNV levels were associated with worse survival in CHOL, GBM, HNSC, LGG, and UCEC, while low CNV levels correlated with better survival in KIRP and LUSC (**Figures 4B, C**). Overall, these results showed *ELFN1* gene mutations, amplifications, and deletions in multiple tumors. Missense mutations were the most frequent type of *ELFN1* gene mutations in various tumors. Moreover, the COAD tissues showed distinct somatic mutations and CNVs based on the expression levels of *ELFN1*. This suggested that alterations in the *ELFN1* gene may regulate the initiation, growth and progression of various tumors, especially COAD. Given the abundance of such mutations in tumors and their potential impact on prognosis and treatment outcomes (37, 38). We also analyzed correlations between *ELFN1* and genomic instability markers, including TMB, MSI, neoantigen load (NEO), ploidy, homologous recombination deficiency (HRD), and loss of heterozygosity (LOH) (**Figures 4E–I**). A negative correlation between *ELFN1* and TMB was detected in MESO, while a positive correlation between *ELFN1* and MSI was observed in TGCT (**Figure 4E**). *ELFN1* was negatively correlated with NEO (**Figures 4F**) in DLBC and with ploidy (**Figures 4G**) in THYM. Conversely, a positive correlation between *ELFN1* and HRD was detected in UCS, MESO, and HNSC (**Figure 4H**). Meanwhile, *ELFN1* was positively correlated with LOH in KIRC, but negatively correlated with LOH in UVM and HNSC (**Figure 4I**). *ELFN1* showed significant associations with these markers, suggesting its role in genomic instability across cancers.

### 3.5 Correlation between ELFN1 levels and DNA repair, methylation, and cancer stemness

**Figure 5:**
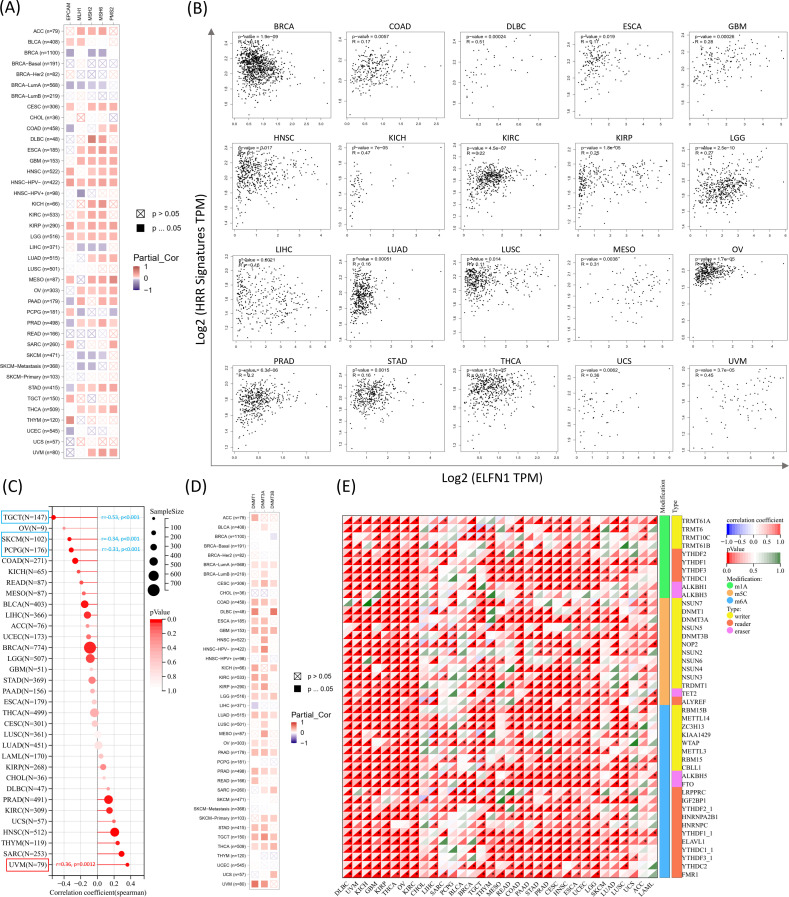
*ELFN1* is associated with DNA repair, epigenetic modifications, and stemness. **(A)** Heatmap showing associations between *ELFN1* and five MMR genes across cancer types. **(B)** Scatter plots highlighting correlations between *ELFN1* expression and a 30-gene HRR signature in 20 cancers. **(C)** Lollipop chart showing correlations between *ELFN1* expression and cancer stemness. **(D)** Heatmap of correlations between *ELFN1* expression and three DNMTs. **(E)** Heatmap showing correlations between *ELFN1* and RNA modification genes across cancers. *P < 0.05, **P < 0.01, ***P < 0.001.

The DNA damage response is a complex mechanism responsible for maintaining genomic stability and integrity by detecting and eliminating abnormal sequences and structures in chromosomes (39). Tumor cells develop mechanisms, such as selecting mismatch repair (MMR) (40) and homologous recombination repair (HRR) (41), to evade certain therapeutic strategies, thereby conferring stemness-like self-renewal capabilities to these tumor cells (42). We next evaluated the association between *ELFN1* expression levels and MMR-related genes, HRR characteristics, and stemness. *ELFN1* was positively correlated with the expression of a series of MMR genes in cancers such as ACC, CESC, DLBC, ESCA, GBM, HNSC-HPV-, KICH, KIRP, LGG, MESO, and OV, while it was negatively correlated in BRCA, LIHC, and SKCM (**Figure 5A**). A positive correlation between *ELFN1* and HRR characteristics was also observed in DLBC, GBM, KICH, KIRP, OV, and UVM, with the strongest correlation in DLBC (**Figure 5B**). *ELFN1* expression was significantly associated with cancer stemness in multiple cancers. Based on the DNAss tumor stemness score, it was positively correlated with stemness in UVM but negatively correlated in TGCT, SKCM, and PCPG (**Figure 5C**). These results suggest that *ELFN1* acts as a regulator of cancer progression by influencing DNA damage repair capabilities. Epigenetic modifications also play a crucial role in shaping cancer development and progression, making them increasingly popular research targets. DNA methyltransferase (DNMT) is responsible for catalyzing DNA methylation, which may regulate tumor cell proliferation, differentiation, survival, and cell cycle progression (43). *ELFN1* expression was significantly positively correlated with DNMT levels in cancers such as CESC, COAD, GBM, LGG, LUAD, PAAD, PRAD, TGCT, and THCA (**Figure 5D**). Notably, we observed that ELFN1 mRNA expression was negatively correlated with methylation in most cancers (**Supplementary Figure S4**). Kaplan-Meier survival analysis showed that reduced methylation predicted shorter survival in ACC, GBM, LGG, SKCM, STAD, THYM, and UVM (**Supplementary Figure S5**). We also used TIDE database to evaluate the relationship between *ELFN1* promoter methylation and cytotoxic T lymphocyte (CTL) levels in cancers such as BLCA, BRCA, BRCA-Luminal A, BRCA-Basal, TNBC, COADREAD, ESCA, GBMLGG, HNSC-HPV+, LUAD, LUSC, PAAD, SARC, STAD, UCEC, and UVM. The results showed a positive correlation in most cancers (**Supplementary Figure S6**). Additionally, we examined the correlation between *ELFN1* and RNA regulatory gene expression (**Figure 5E**). In summary, these analyses indicate that *ELFN1* plays an important role in DNA methylation and mRNA modifications across various cancer types.

### 3.6 Relationship between ELFN1 expression, tumor immune microenvironment, and immunotherapy

**Figure 6:**
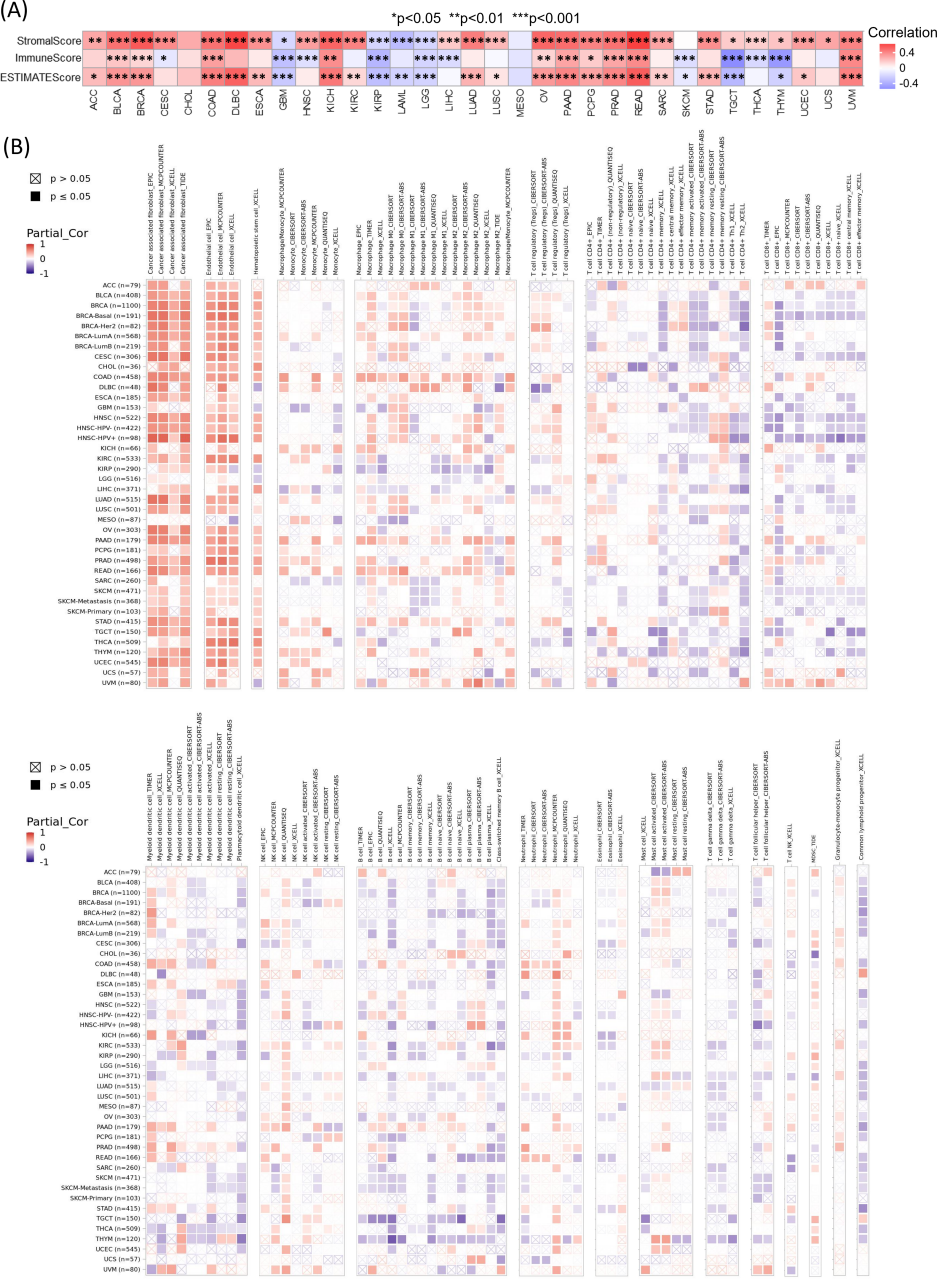
Relationship between *ELFN1* expression and TIME. **(A)** Heatmap showing correlations between *ELFN1* expression and StromalScore, ImmuneScore, and ESTIMATEScore across cancer types. **(B)** Immune cell infiltration analysis of *ELFN1* in TCGA cancers using the TIMER2.0 portal. *P < 0.05, **P < 0.01, and ***P < 0.001.

We further investigated the potential immunoregulatory functions of *ELFN1* in tumor immunity. The ESTIMATE algorithm was used to study the correlation between *ELFN1* expression and the TIME in different cancer types. ESTIMATE analysis showed that *ELFN1* expression was significantly positively correlated with immune and stromal scores in cancers such as BLCA, BRCA, COAD, KICH, OV, PAAD, PCPG, PRAD, READ, and UVM, while it was negatively correlated in GBM, KIRP, and LGG (**Figure 6A**). The six cancers with the strongest correlations are shown in **Supplementary Figure S7**. Using the TIMER2.0 database, we further evaluated the correlation between *ELFN1* and various immune-related cells in different cancers, including CAFs, endothelial cells, macrophages, Treg cells, CD4+ memory T cells, CD8+ T cells, B cells, and neutrophils, using algorithms such as EPIC, TIMER, CIBORSORT, and xCELL. *ELFN1* was found to be significantly positively correlated with CAFs and endothelial cells in most cancers (**Figure 6B**; **Supplementary Figure S8**).

**Figure 7:**
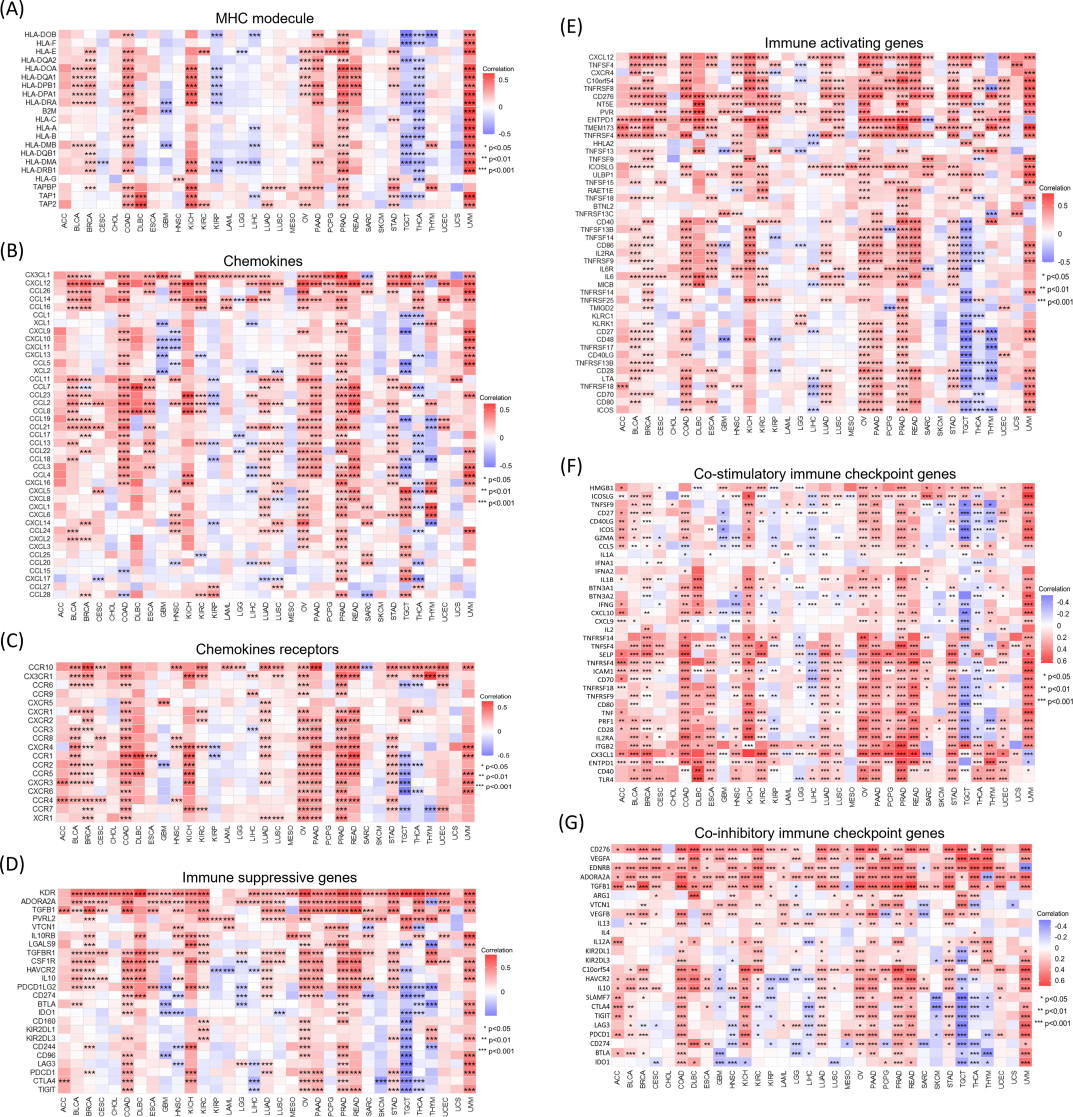
Correlations between *ELFN1* expression and immune-related genes. Correlations between *ELFN1* expression and **(A)** MHC genes, **(B)** chemokines, **(C)** chemokine receptors, **(D)** immunosuppressive genes, **(E)** immune-activating genes, **(F)** co-stimulatory immune checkpoint genes, and **(G)** co-inhibitory immune checkpoint genes. *P < 0.05, **P < 0.01, ***P < 0.001.

We also performed pan-cancer analysis of the association between *ELFN1* and immunoregulatory genes as well as immune checkpoint genes (**Figure 7**). The results revealed significant correlations between *ELFN1* and immune regulation in various cancers, suggesting that *ELFN1* may have diverse immune functions across cancers. To further explore the potential of *ELFN1* as a target for cancer immunotherapy, we analyzed the OS of patients with high or low *ELFN1* expression who received anti-PD1, anti-PDL1, or anti-CTLA4 therapy. The results showed that patients with high *ELFN1* expression had better OS compared to those with low *ELFN1* expression (**Supplementary Figure S9A**). Specifically, patients with high *ELFN1* expression who received anti-PD-L1 therapy (**Supplementary Figures S9E, F**) showed significantly improved OS compared to those with low *ELFN1* expression. However, patients with high *ELFN1* expression who received anti-PD1 therapy (**Supplementary Figures S9B-D**) or anti-CTLA4 therapy (**Supplementary Figure S9G**) exhibited resistance to treatment, with significantly reduced OS. These findings have important implications for personalized medicine, as determining *ELFN1* expression levels could help physicians select the most appropriate immunotherapy, thereby improving patient survival and treatment outcomes.

### 3.7 Single-cell functional analysis of ELFN1

**Figure 8:**
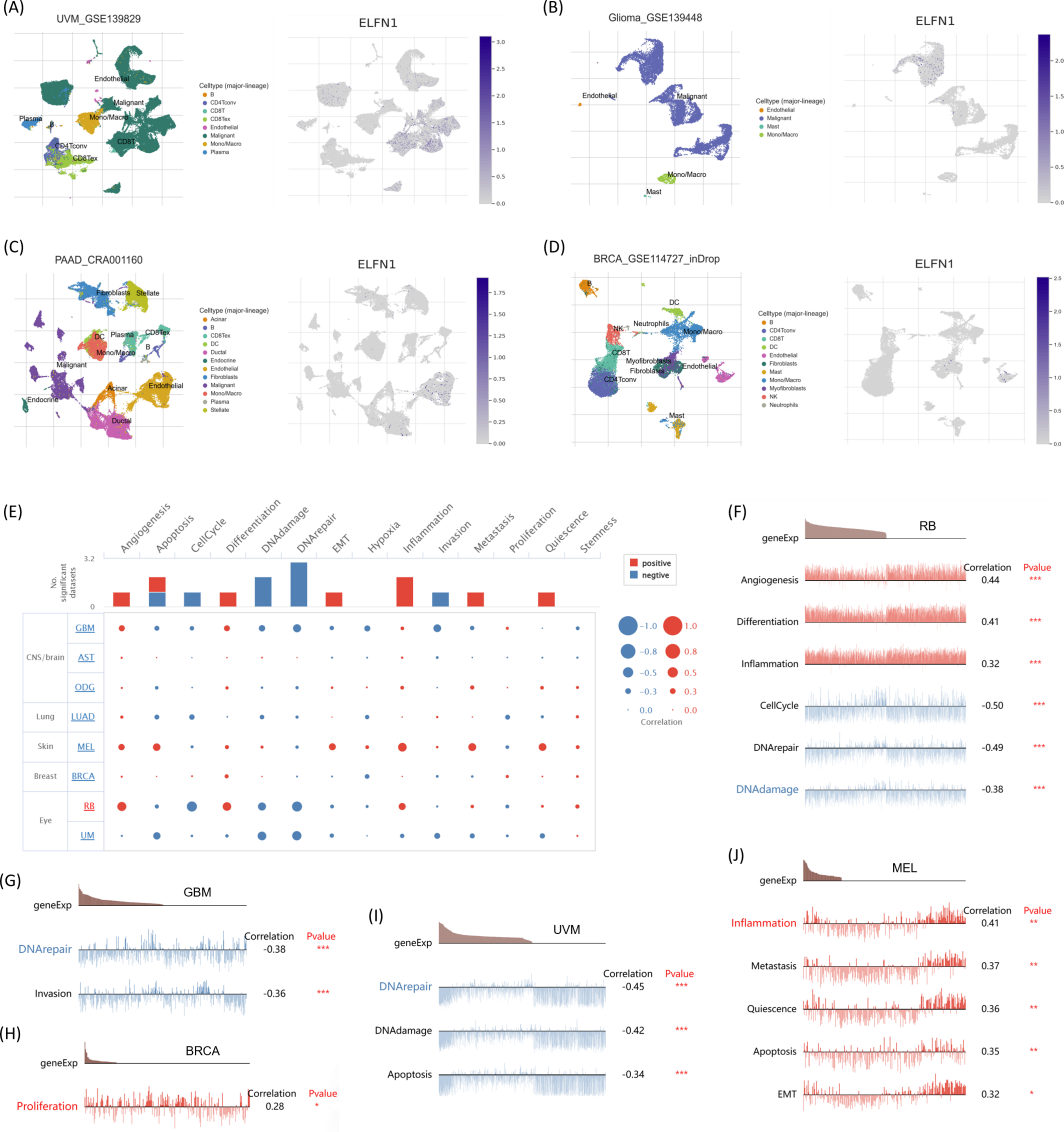
Single-cell analysis of *ELFN1* in human cancers. **(A–D)** Composition and distribution of single cells expressing *ELFN1* in UVM, glioma, PAAD, and BRCA from the TISCH database, ranked by average expression. **(E)** Functional status of *ELFN1* across cancers from the CancerSEA database. **(F–J)** Correlation analysis between *ELFN1* expression and functional status in RB, GBM, BRCA, UVM, and MEL. *P < 0.05, **P < 0.01, ***P < 0.001.

To better understand the main cell types expressing *ELFN1* in the tumor microenvironment, we performed single-cell analysis of *ELFN1* expression across 79 tumor single-cell datasets. Data from the TISCH database showed *ELFN1* expression levels in each cell type (including immune cells, stromal cells, malignant cells, and functional cells) in the single-cell datasets. The results revealed that *ELFN1* is primarily expressed in immune cells (especially endothelial cells and fibroblasts) and malignant cells in tumors. In the UVM GSE139829 dataset (**Figure 8A**) and the Glioma GSE139448 dataset (**Figure 8B**), *ELFN1* was mainly expressed in malignant cells, with low expression in endothelial cells and fibroblasts. In contrast, in the PAAD CRA001160 dataset (**Figure 8C**) and the BRCA GSE114727_inDrop dataset (**Figure 8D**), *ELFN1* was primarily expressed in endothelial cells and fibroblasts, consistent with our previous predictions regarding the TME. To further investigate the potential role of *ELFN1* in tumors, we used the CancerSEA database to study the function of *ELFN1* at the single-cell level (**Figure 8E**). The results showed that *ELFN1* is positively correlated with angiogenesis, differentiation, and inflammation in RB, while negatively correlated with the cell cycle, DNA repair, and DNA damage (**Figure 8F**). In GBM, *ELFN1* is negatively correlated with DNA repair and invasion (**Figure 8G**). In BRCA, *ELFN1* is positively correlated with proliferation (**Figure 8H**). In UVM, *ELFN1* is negatively correlated with DNA repair, DNA damage, and apoptosis (**Figure 8I**). In MEL, *ELFN1* is positively correlated with angiogenesis, metastasis, quiescence, apoptosis, and epithelial-mesenchymal transition (**Figure 8J**).

### 3.8 Protein-protein interaction network and enrichment analysis of ELFN1

**Figure 9:**
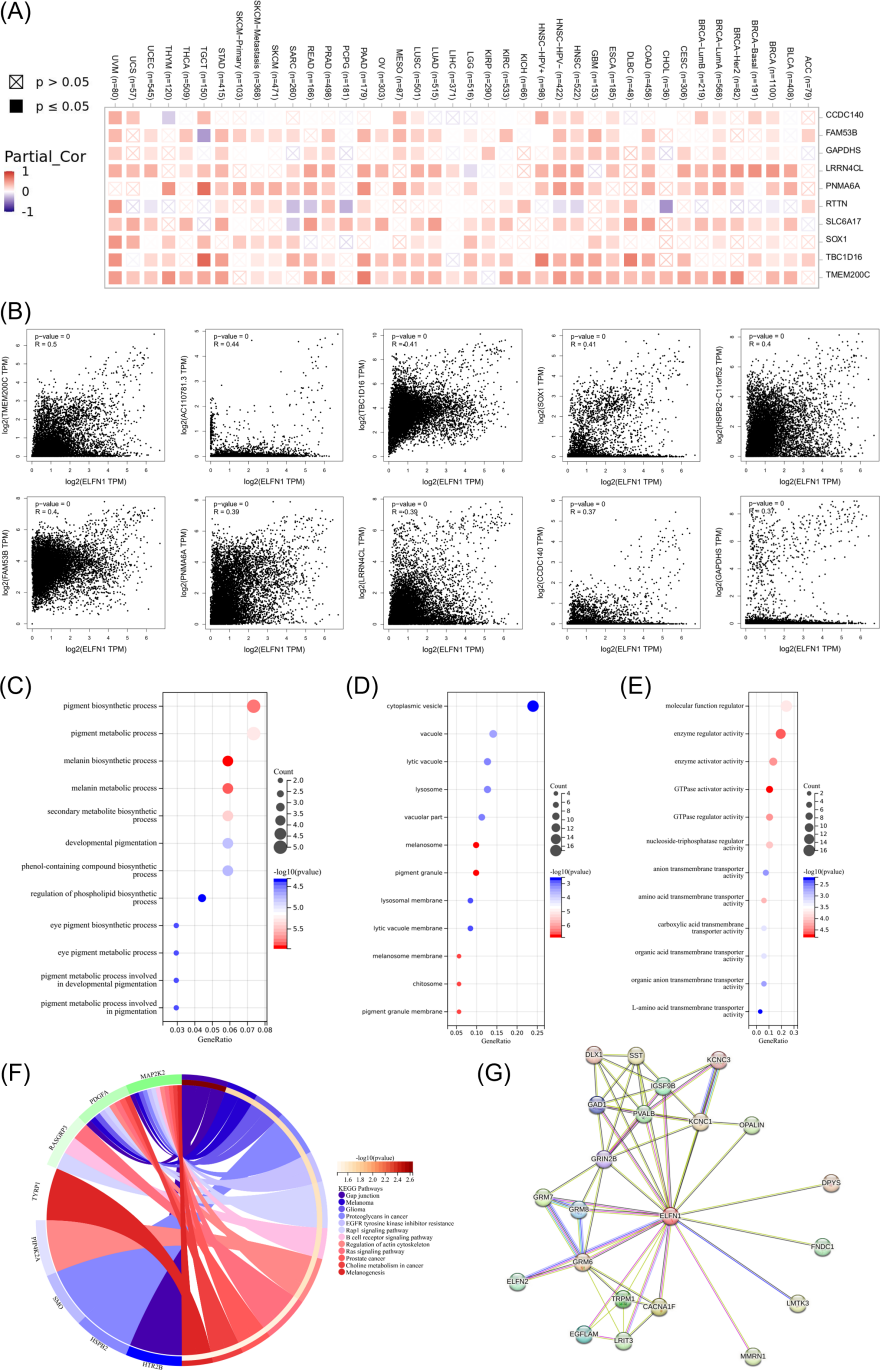
*ELFN1*-related genes enrichment analysis. **(A)** Heatmap and **(B)** scatter plots of the top 10 *ELFN1*-related genes in cancers. **(C–E)** GO enrichment analysis for BP **(C)**, CC **(D)**, and MF **(E)** based on *ELFN1*-interacted and correlated genes. **(F)** KEGG pathway analysis of *ELFN1*-interacted and correlated genes. **(G)** Predicted *ELFN1*-interacting proteins from the STRING database.

We utilized the STRING database to construct the PPI network of ELFN1 (**Figure 9G**). Using the GEPIA2.0 database, we identified the top 100 genes associated with *ELFN1* (**Figures 9A, B**; **Supplementary Table S2**) and performed Gene Ontology (GO) and KEGG enrichment analyses on these genes (**Supplementary Table S3**). In the biological process (BP) enrichment analysis, we found that *ELFN1*-related genes are involved in pigmentation and pigment regulation (**Figure 9C**). The cellular component (CC) enrichment analysis showed that *ELFN1* is primarily enriched in cytoplasmic vesicles, lysosomes, melanosomes, and pigment granules (**Figure 9D**). The molecular function (MF) enrichment analysis revealed that *ELFN1* is associated with kinase regulatory activity and transmembrane transporter activity, suggesting its role in tumor pathogenesis (**Figure 9E**). Additionally, KEGG pathway analysis indicated that *ELFN1* participates in multiple signaling pathways, including gap junctions, melanoma-related pathways, glioma-related pathways, proteoglycans in cancer, EGF tyrosine kinase inhibitor resistance, Rap1 signaling pathway, B cell receptor signaling pathway, actin cytoskeleton regulation, Ras signaling pathway, prostate cancer-related pathways, choline metabolism in cancer, and melanogenesis pathways (**Figure 9F**). These results indirectly reveal the molecular mechanisms by which *ELFN1* contributes to tumorigenesis.

### 3.9 Drug sensitivity analysis of ELFN1

**Figure 10:**
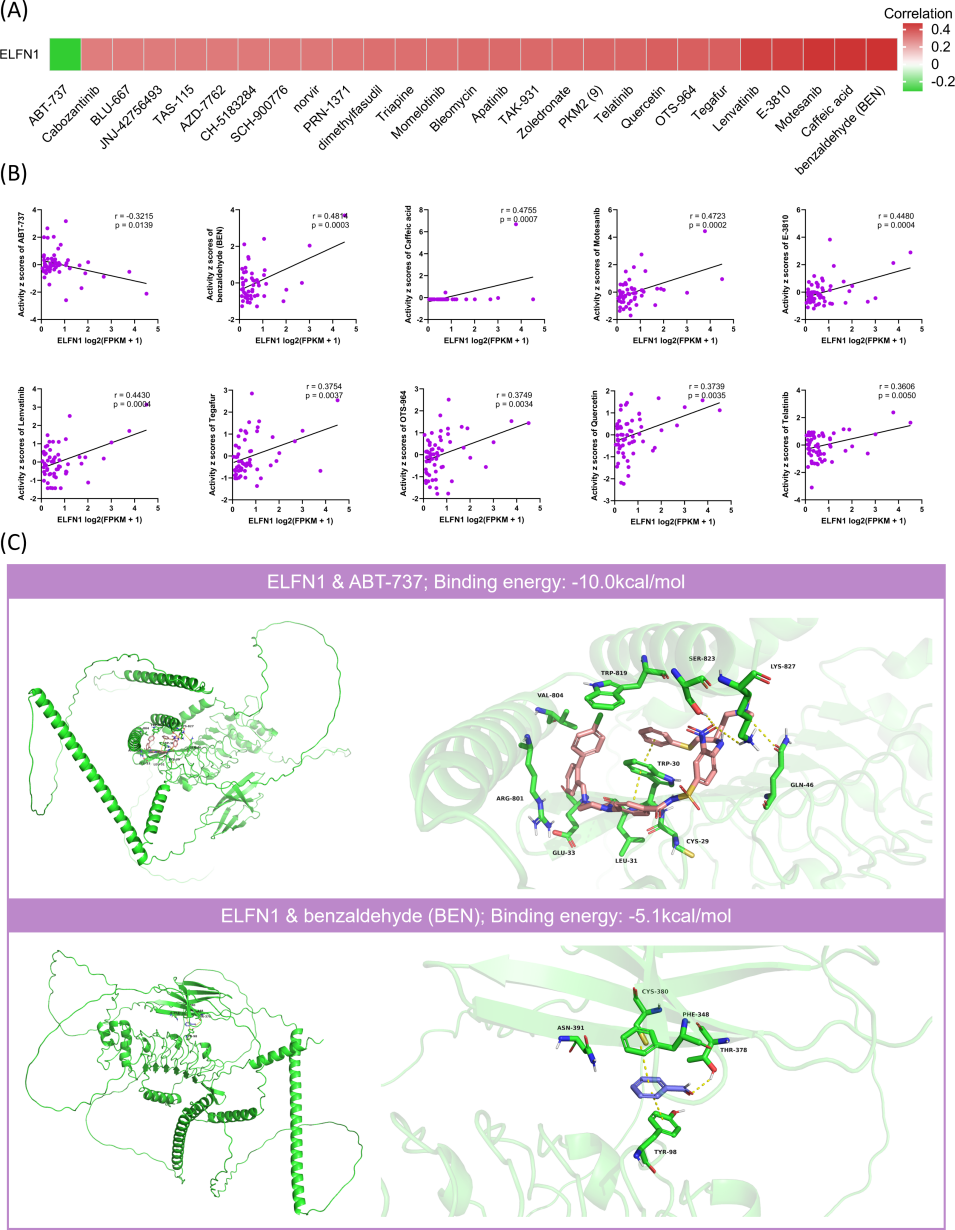
*ELFN1* is associated with drug sensitivity. **(A)** Correlation between *ELFN1* expression and IC50 values of drugs from the CellMiner database. **(B)** Scatter plots of the top 10 drugs most correlated with *ELFN1* expression. **(C)** Molecular docking between *ELFN1* protein and drugs with the strongest negative (upper) and positive (lower) correlations. CYS, cysteine; TRP, tryptophan; LEU, leucine; GLU, glutamic acid; GLN, glutamine; ARG, arginine; VAL, valine; SER, serine; LYS, lysine; TYR, tyrosine; PHE, phenylalanine; THR, threonine; ASN, asparagine.

Enhancing drug sensitivity is critical for preventing cancer cells from developing resistance to treatment. To further explore this, we analyzed the correlation between *ELFN1* expression levels and drug sensitivity using data from the CellMiner database. ELFN1 was significantly correlated with the sensitivity of 27 FDA-approved or Clinical trial drugs (absolute correlation coefficient |Cor| > 0.3) (**Figures 10A, B**). Specifically, *ELFN1* expression was negatively correlated with the sensitivity to ABT-737, while it was positively correlated with the sensitivity to 26 other drugs, including benzaldehyde (BEN), caffeic acid, motesanib, E-3810, and lenvatinib (Cor > 0.4). This suggests that patients with high *ELFN1* expression may be particularly sensitive to ABT-737, while showing resistance to drugs such as benzaldehyde (BEN), caffeic acid, motesanib, E-3810, and lenvatinib. To further evaluate the binding affinity between ELFN1 and the candidate drugs, we performed molecular docking analyses. Results indicated strong binding interactions between ELFN1 and these compounds (**Supplementary Table S4**). Representative molecular docking poses are shown for ELFN1 bound to the most sensitive drug, ABT-737 (Cor = −0.3215), and the most resistant drug, benzaldehyde (BEN) (Cor = 0.4814) (**Figure 10C**).

Additionally, we used the GSCA database to explore the relationship between *ELFN1* and chemotherapy drug sensitivity. Analysis using the Cancer Therapeutics Response Portal (CTRP) database revealed a negative correlation between *ELFN1* levels and the IC50 values of multiple compounds, including GSK-J4, PF-3758309, omacetaxine mepesuccinate, brivanib, BRD-K35604418, oligomycin A, axitinib, and valdecoxib (**Supplementary Figure S10A**). Similarly, analysis based on the Genomics of Drug Sensitivity in Cancer (GDSC) database showed a negative correlation between *ELFN1* levels and the IC50 values of axitinib, pazopanib, elesclomol, YK 4-279, TW 37, midostaurin, and bleomycin (50 μM) (**Supplementary Figure S10B**). It is noteworthy that the largest proportion of these candidate drugs were found to be kinase inhibitors. These findings suggest that *ELFN1* is associated with sensitivity to multiple drugs, making it a promising target for chemotherapy.

### 3.10 Silencing ELFN1 expression inhibits the proliferation, motility, and migration of CRC cells

The initial pan-cancer analysis demonstrated increased expression of ELFN1 across 18 tumor types, including ALL, BLCA, COAD, and others. Notably, elevated ELFN1 expression was significantly associated with poorer overall survival in UVM, COAD, CESC, LUAD, and SKCM. Through an integrated analysis of differential expression and prognostic data, COAD and SKCM emerged as the most pertinent cancer types, with ELFN1 showing the highest hazard ratio in COAD. Consequently, we selected CRC cell lines for subsequent experimental validation.

**Figure 11:**
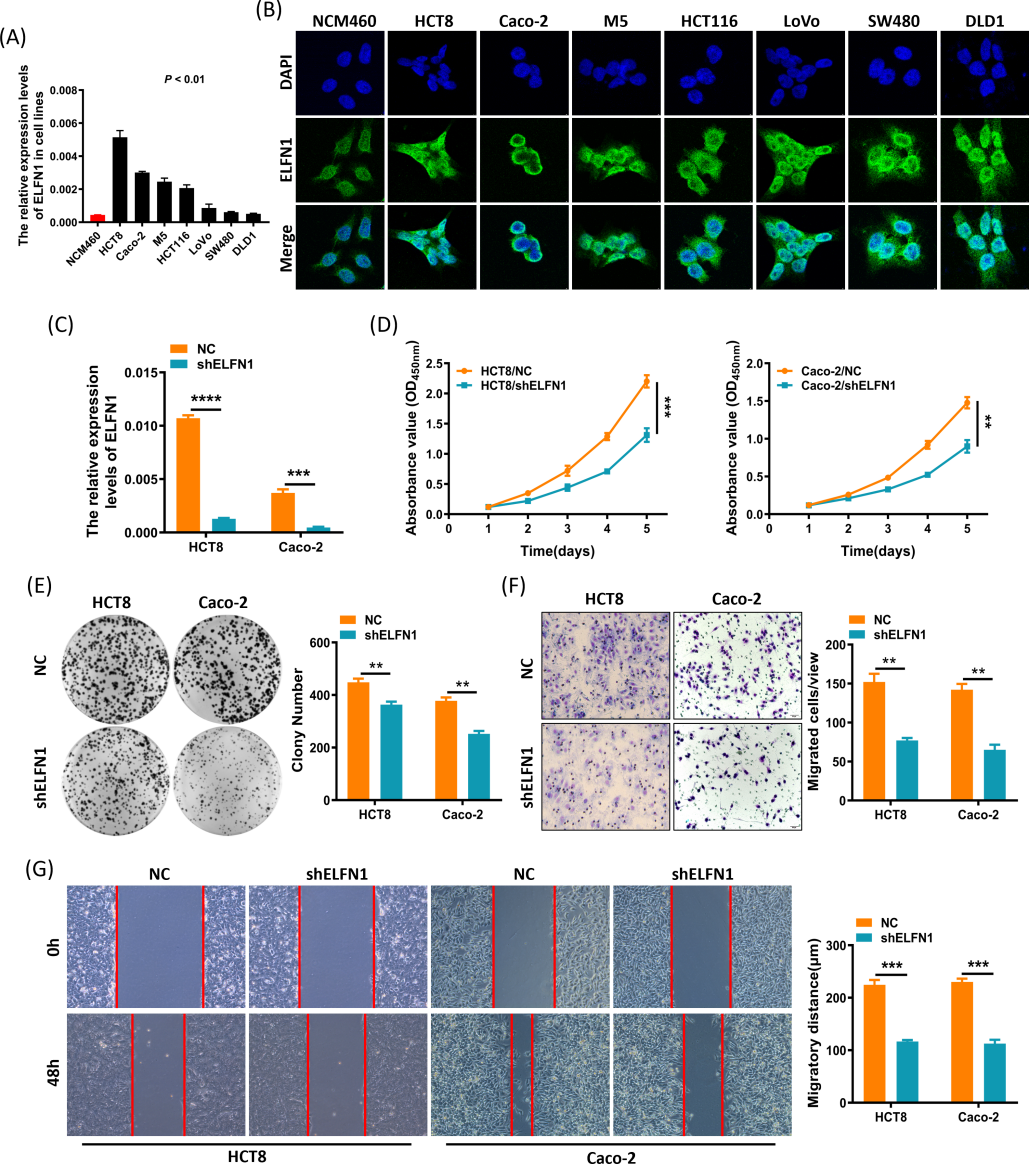
*ELFN1* is upregulated in CRC cell lines and *ELFN1* depletion suppresses CRC cell proliferation, migration, and invasion *in vitro*. **(A)***ELFN1* mRNA expression in normal colorectal cells (NCM460) and CRC cells. **(B)** Immunofluorescence showing *ELFN1* expression and localization in NCM460 and CRC cells. **(C)***ELFN1* mRNA levels in shRNA-transfected cells. **(D, E)** Effects of *ELFN1* knockdown on CRC cell proliferation using CCK-8 **(D)** and colony formation assays (**E**, representative images on the left, quantitative results on the right). **(F)** Transwell assays showing *ELFN1* depletion effects on CRC cell invasion (representative images on the left, quantitative results on the right). **(G)** Wound healing assays showing *ELFN1* knockdown effects on migration (representative images on the left, quantitative results on the right). Data are presented as means ± SEM from three independent experiments. *P < 0.05, **P < 0.01, ***P < 0.001, ****P < 0.0001.

To further confirm the relationship between *ELFN1* and colorectal cancer, we examined the expression status of ELFN1 in CRC cells. We found that *ELFN1* mRNA expression levels were higher in CRC cell lines compared to normal colorectal cells (NCM460) (**Figure 11A**). Immunofluorescence analysis revealed that ELFN1 is localized in both the nucleus and cytoplasm of CRC cell lines and NCM460 cells (**Figure 11B**). Given the high expression of *ELFN1* in CRC cells, we transfected CRC cell lines HCT8 and Caco-2 with sh-NC (negative control) and sh-ELFN1 (*ELFN1* knockdown) (**Figure 11C**) and evaluated their effects on cell proliferation, motility, and migration. CCK8 assays showed that sh-ELFN1 significantly inhibited the proliferation of HCT8 and Caco-2 cells (**Figure 11D**). Similarly, colony formation assays revealed a significant reduction in the number of cell colonies in the sh-ELFN1 group compared to the sh-NC group (**Figure 11E**). Transwell assays demonstrated that the migration ability of HCT8 and Caco-2 cells was significantly suppressed in the sh-ELFN1 group compared to the sh-NC group (**Figure 11F**). Additionally, wound healing assays showed that sh-ELFN1 significantly inhibited the migration ability of HCT8 and Caco-2 cells (**Figure 11G**). These findings indicate that silencing ELFN1 expression leads to significant inhibition of the proliferation, motility, and migration of CRC cell lines HCT8 and Caco-2.

## 4 Discussion

The global incidence and mortality rates of cancer continue to rise annually, posing a severe threat to public health. Despite advancements in cancer treatment, the prognosis and survival rates of patients remain unsatisfactory due to challenges such as drug resistance, adverse side effects, and other complications (44). Investigating the relationship between specific genes and immunotherapy could provide a robust theoretical foundation for developing targeted therapies and improving immunotherapy outcomes in cancer patients.

Recent studies indicate that ELFN1 may have broad functional roles in diverse biological processes, including immune regulation and cancer progression. Elevated ELFN1 expression is strongly associated with poor progression-free survival (PFS), overall survival (OS), and resistance to tumor-infiltrating lymphocyte (TIL) therapy in metastatic melanoma patients (14). Supporting its role in TIL resistance, enhanced ELFN1 methylation in baseline tumor tissues correlates with treatment response in these patients (14). In colorectal cancer (CRC), ELFN1 has been identified as a potential neutrophil extracellular trap (NET)-associated differentially expressed gene (DEG) in prognostic models and exhibits positive correlation with the NETs signaling pathway (17). Given the established link between NETs and the immune microenvironment (45) and the potential of immune cell infiltration as a predictive indicator in CRC (46). NETs_high samples exhibited an enriched immune environment, potentially influencing CRC prognosis (17). Furthermore, ELFN1 expression has been documented in other cancers, such as breast and ovarian cancers (15, 16). with downregulation observed in breast cancers exhibiting specific histologic features including inflammation, necrosis, pronounced nuclear pleomorphism, and moderate/high mitotic counts (16). Building on these findings, this study systematically analyzed the expression, prognostic significance, and functional roles of *ELFN1* across multiple cancer types. *ELFN1* was found to be upregulated in most cancers and exhibited high diagnostic value. Dysregulated *ELFN1* expression in certain cancers was associated with abnormal copy number variations and methylation patterns. Furthermore, *ELFN1* was linked to poor prognosis in several cancers, suggesting its potential as a prognostic biomarker. Functional studies also revealed that *ELFN1* promotes the progression of CRC. In agreement with a recent study (47), our *in vitro* experiments confirmed that ELFN1 promotes the proliferation, motility, and migration of colorectal cancer (CRC) cells.

This study aimed to explore the role of *ELFN1* in the initiation and progression of various cancers. To achieve this, expression analyses were conducted. In normal human tissues, *ELFN1* was highly expressed in liver tissues, while single-cell sequencing of healthy tissues revealed that *ELFN1* was predominantly enriched in fibroblasts and endothelial cells, indicating its potential involvement in microenvironmental regulation. Comparisons between normal and tumor tissues showed that *ELFN1* expression was dysregulated in most tumor types and was associated with various clinical factors, underscoring its prognostic value in cancers such as CESC, COAD, KIRC, LIHC, LUAD, SKCM, and UVM. Notably, high *ELFN1* expression was identified as a marker of poor prognosis in COAD.

Somatic mutations in key genes are known to drive the transformation of normal cells into cancer cells (48). These mutations also contribute to immune evasion and poor therapeutic responses (49). In this study, amplifications, mutations, and deep deletions were identified as the most frequent alterations in the *ELFN1* gene across several cancer types. Survival analysis revealed that tumors with ELFN1 mutations had worse outcomes compared to those with wild-type *ELFN1*. This may be attributed to the impact of *ELFN1* alterations on transcriptional regulation and post-translational functional roles.

Immunotherapy, particularly ICB, has revolutionized cancer treatment, offering significant clinical benefits across various tumor types (20). However, the efficacy of immunotherapy varies among patients with the same cancer type, likely due to differences in the immune characteristics of tumors (50, 51). Tumors with high MSI (MSI-H), TMB, or neoantigens have been shown to respond better to immunotherapy (52). In this study, *ELFN1* was found to correlate with TMB and MSI in certain tumor types, though the correlations were not highly significant. Analysis of patients undergoing different immune therapies revealed that high *ELFN1* expression was associated with improved overall survival in some cases. For example, patients undergoing comprehensive anti-PD-L1 therapy (such as Atezolizumab) exhibited improved survival outcomes with elevated ELFN1 expression, and patients treated with exclusive anti-PD1 therapies (such as Pembrolizumab or Nivolumab) or anti-CTLA4 therapy (such as Ipilimumab) demonstrated enhanced survival outcomes with reduced ELFN1 expression. These findings highlight the importance of considering *ELFN1* expression levels when designing personalized immunotherapy strategies to optimize treatment efficacy.

ELFN1 is an allosteric modulator of type III metabotropic glutamate receptors (mGluR7 and mGluR6) (13, 53). While type I and II metabotropic glutamate receptors are known to modulate immune responses and are expressed in T cells, type III receptors have not been reported in T cells (54). Since ELFN1 does not interact with type I or II receptors, it is unlikely to directly regulate T cell function (13). Instead, studies have shown that *ELFN1* is expressed in CAFs and endothelial cells in various tumor tissues (14, 18). Consistent with these findings, this study demonstrated a significant positive correlation between *ELFN1* expression and the presence of CAFs and endothelial cells in most tumors.

The TME plays a critical role in determining the success of immunotherapy in eradicating cancer cells. Effective anti-tumor immune responses rely on the activation, mobilization, infiltration, and elimination of tumor cells by effector T cells (55). CAFs are key components of the TME, contributing to tumor progression by producing growth factors, cytokines, extracellular matrix (ECM) proteins (e.g., collagen and fibronectin), and matrix metalloproteinases (MMPs) (56). Tumor vasculature is often characterized by incomplete development and increased permeability, along with stromal components such as fibroblasts and ECM, which collectively create barriers that hinder T cell infiltration (57, 58). These TME components regulate T cell movement and infiltration through complex mechanisms, leading to immune evasion and the formation of “cold” tumors (20). The evidence suggests that *ELFN1* may influence the TME by modulating the activity of CAFs and endothelial cells, thereby affecting the efficacy of immunotherapy.

GO and KEGG pathway analyses revealed that *ELFN1* is involved in regulating multiple tumor-associated pathways, including those related to enzyme and GTPase activities. Previous research has shown that the GTPase activator RGS1 in tumor-specific circulating T cells suppresses chemokine receptor signaling, reducing T cell motility and infiltration of CTL and Th1 cells in mouse models, breast cancer, and lung cancer (59). These findings suggest that *ELFN1* may also influence immunomodulation by regulating GTPase activity. Our systematic analysis highlights the characterization of *ELFN1* in cancer tissues and identifies its potential as an important prognostic biomarker and immunotherapeutic target for specific cancer types. Valuable insights were provided to understand the role of *ELFN1* in malignant tumors.

## 5 Conclusions

This study demonstrates that *ELFN1* is aberrantly expressed in various cancers and is significantly associated with patient prognosis. Alterations in the *ELFN1* gene, including mutations, amplifications, and deletions, were identified across multiple cancer types. *ELFN1* expression was strongly correlated with CAFs in the TME and the response to immunotherapy in several cancers. *In vitro* experiments confirmed that *ELFN1* functions as an oncogene in CRC. These findings suggest that *ELFN1* is a promising prognostic biomarker and a potential therapeutic target for improving immunotherapy outcomes. However, this discovery requires further *in vitro* and *in vivo* experimental exploration to elucidate the specific mechanisms by which ELFN1 functions during tumor progression and immunotherapy, which is crucial for advancing immune-based therapeutic strategies for tumors.

## Data Availability

The original contributions presented in the study are included in the article/**Supplementary Material**. Further inquiries can be directed to the corresponding author/s.
