# Supplementary figures and images for: Pan-cancer analysis reveals ELFN1 as a novel prognostic biomarker and immunotherapeutic target associated with tumor microenvironment remodeling and promoting malignant phenotypes in colorectal cancer
Source: Front Oncol. 2025 Nov 20;15:1583277. doi: 10.3389/fonc.2025.1583277 (PMC12675275; doi:10.3389/fonc.2025.1583277)

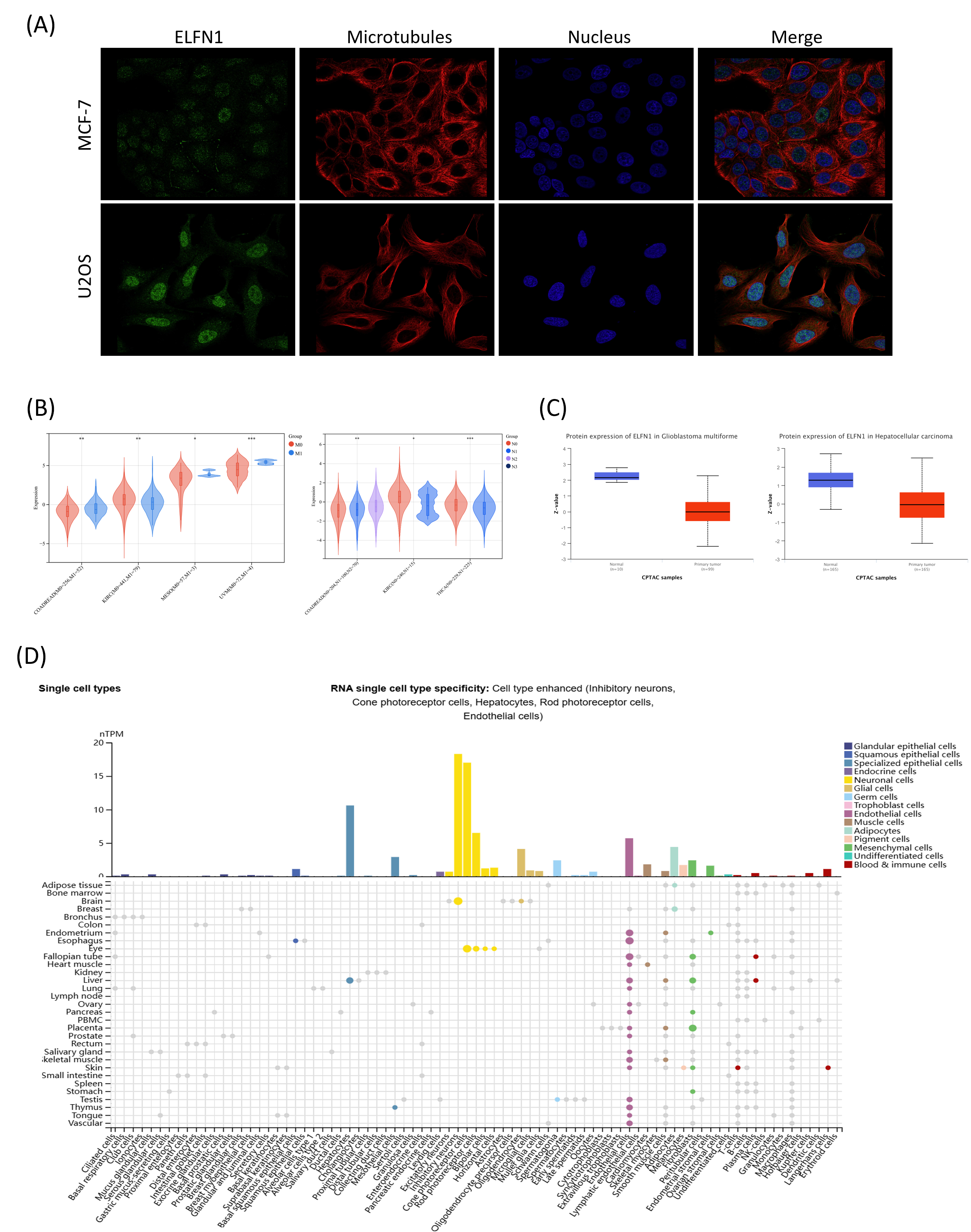

Supplement: Supplementary file 1 [file Image1.tif]

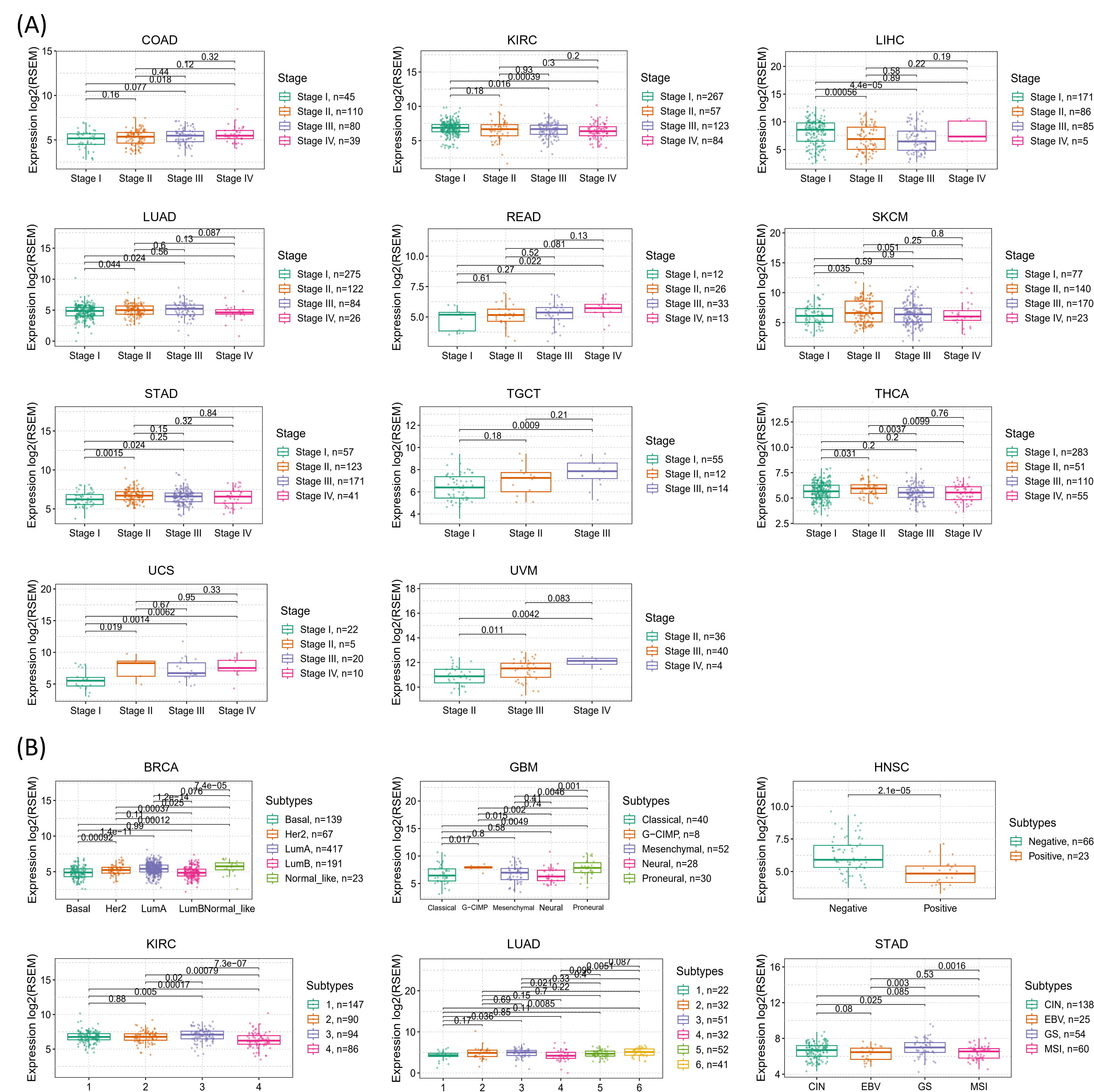

Supplement: Supplementary file 2 [file Image2.tif]

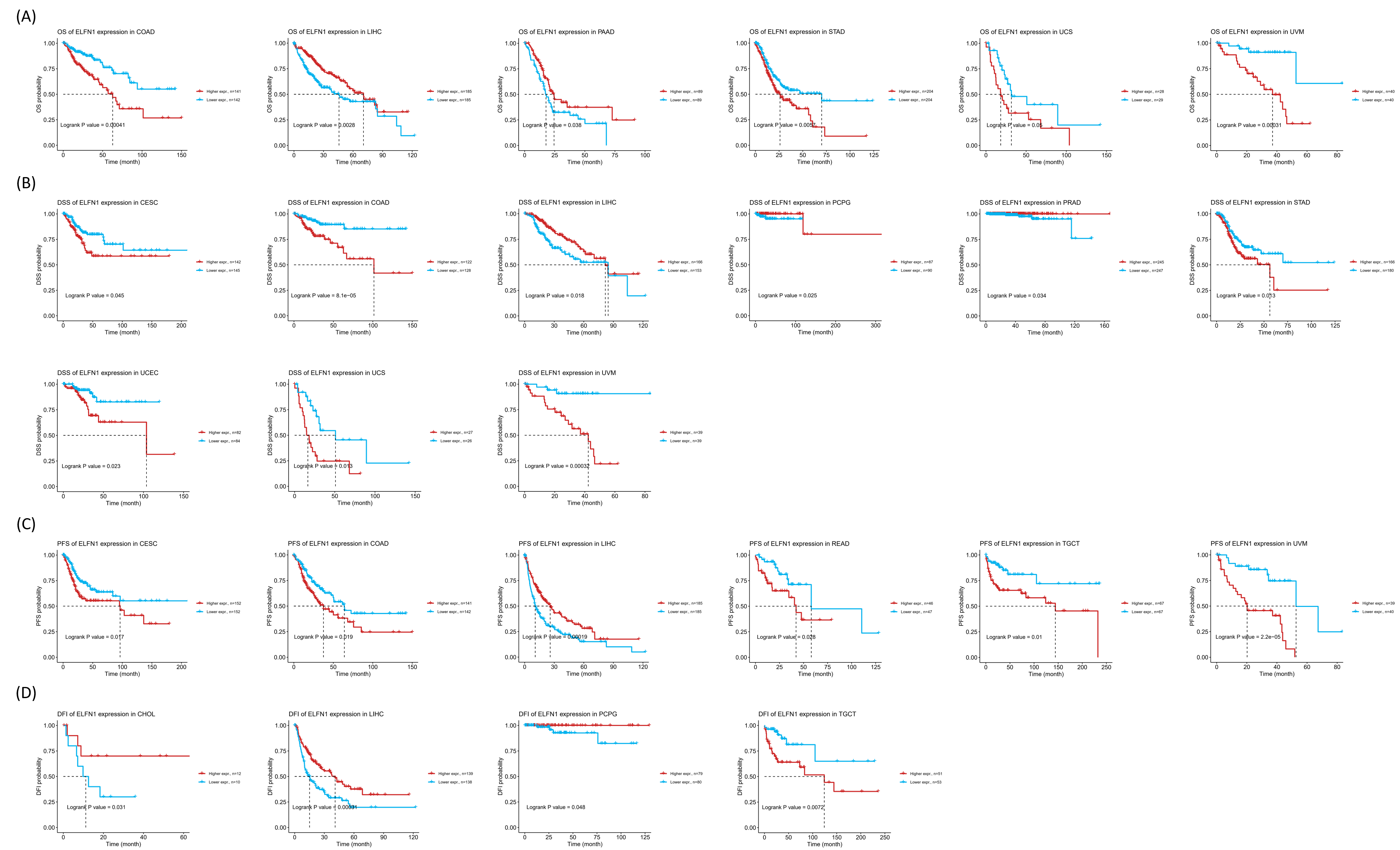

Supplement: Supplementary file 3 [file Image3.tif]

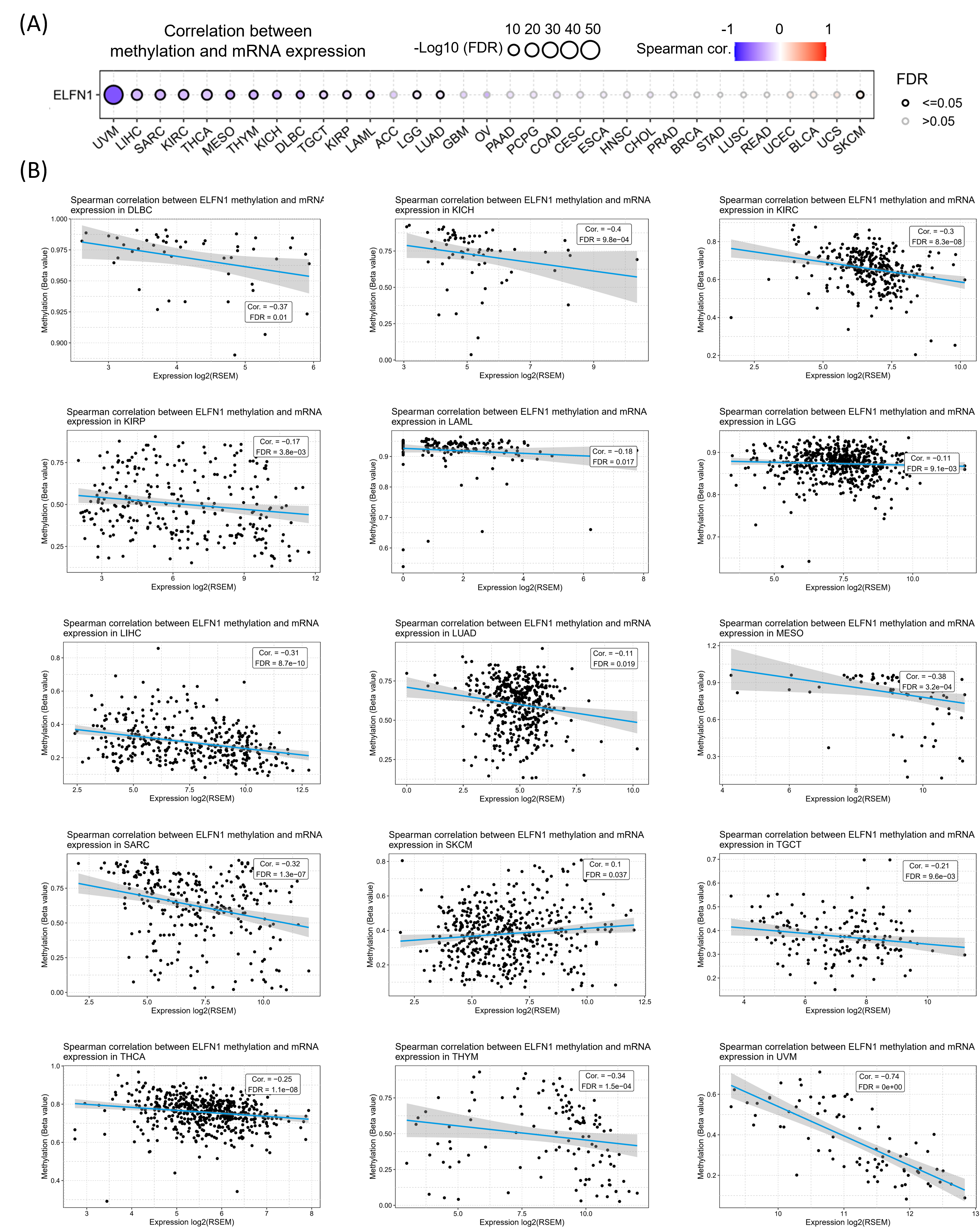

Supplement: Supplementary file 4 [file Image4.tif]

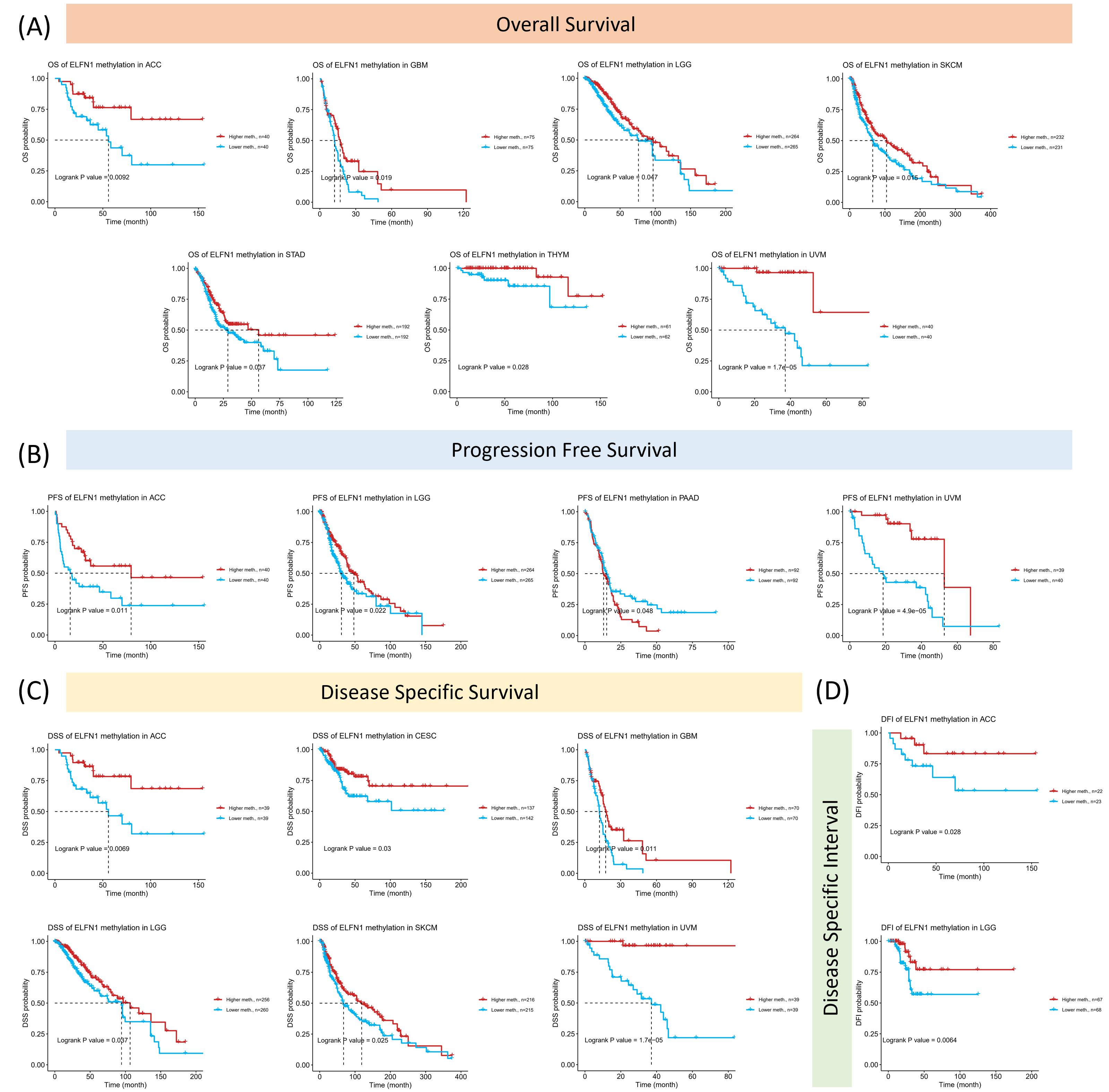

Supplement: Supplementary file 5 [file Image5.tif]

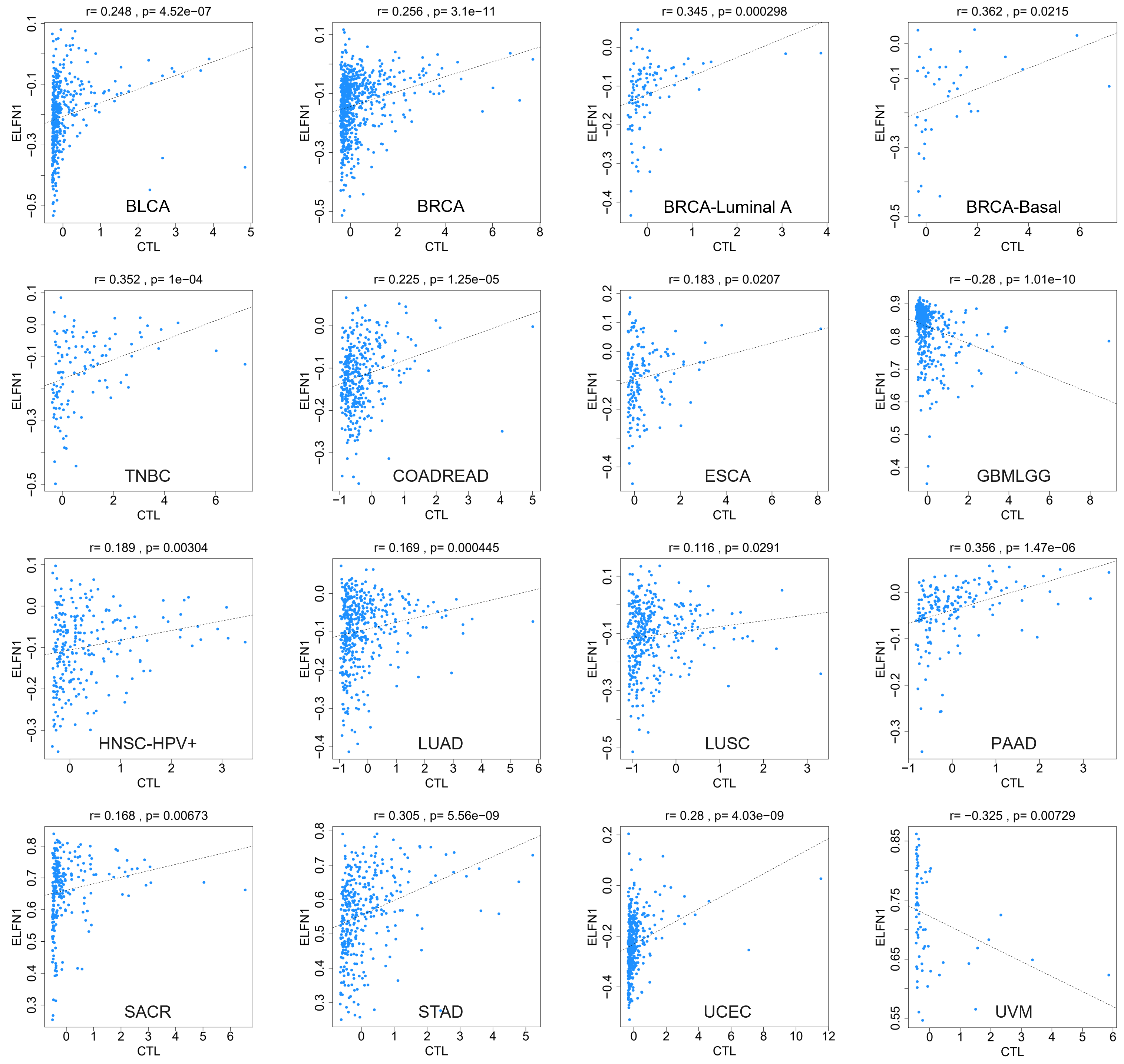

Supplement: Supplementary file 6 [file Image6.tif]

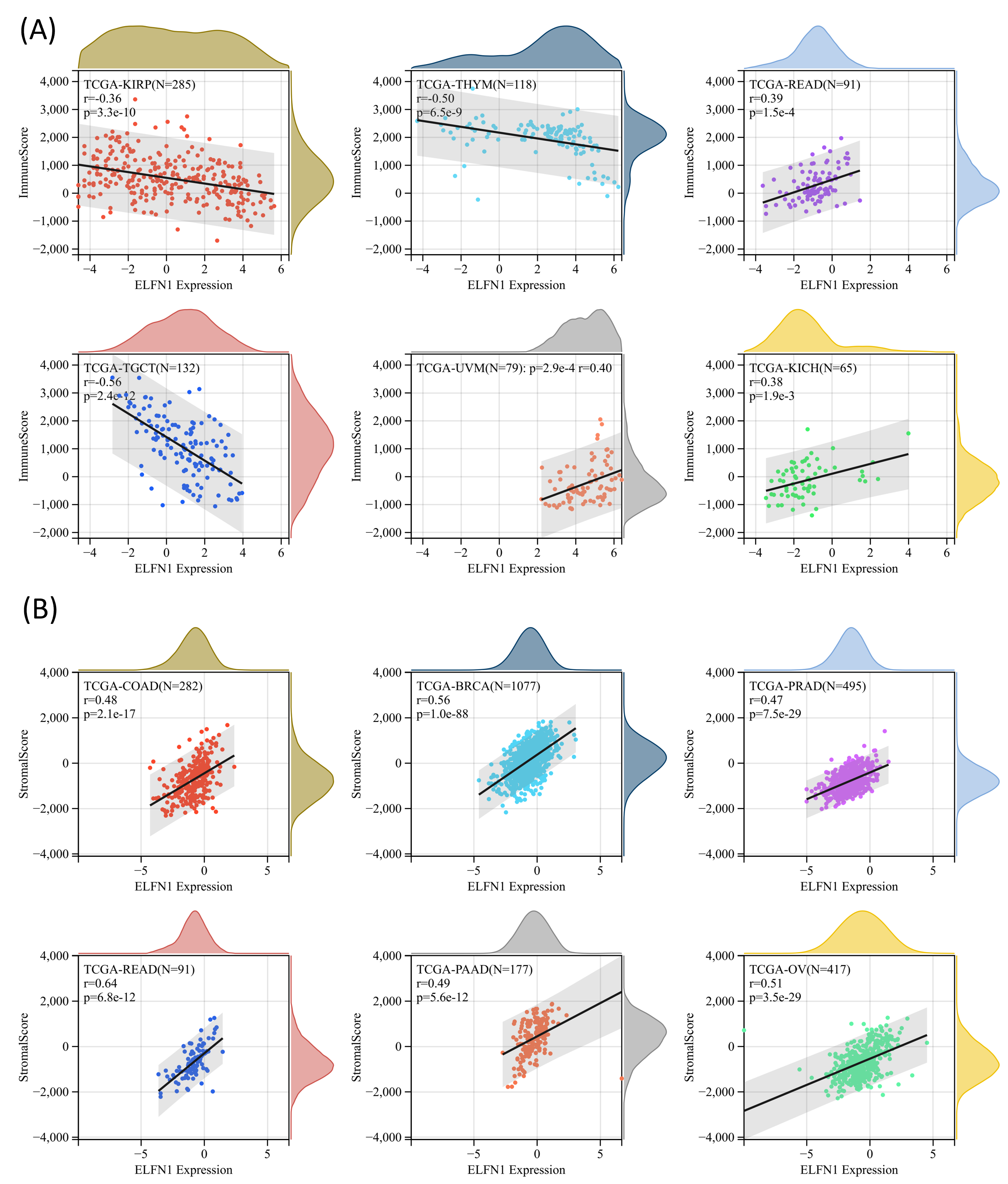

Supplement: Supplementary file 7 [file Image7.tif]

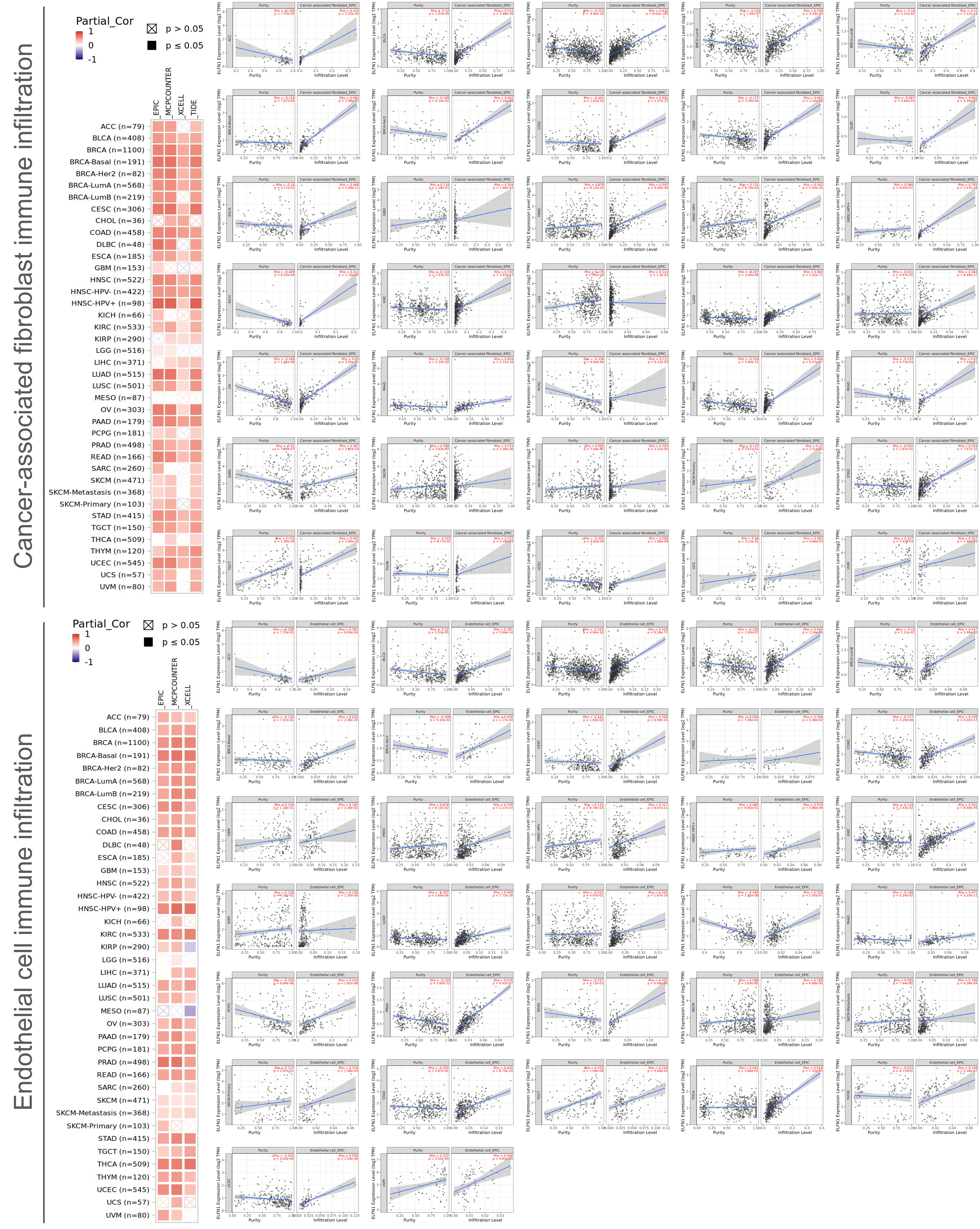

Supplement: Supplementary file 8 [file Image8.tif]

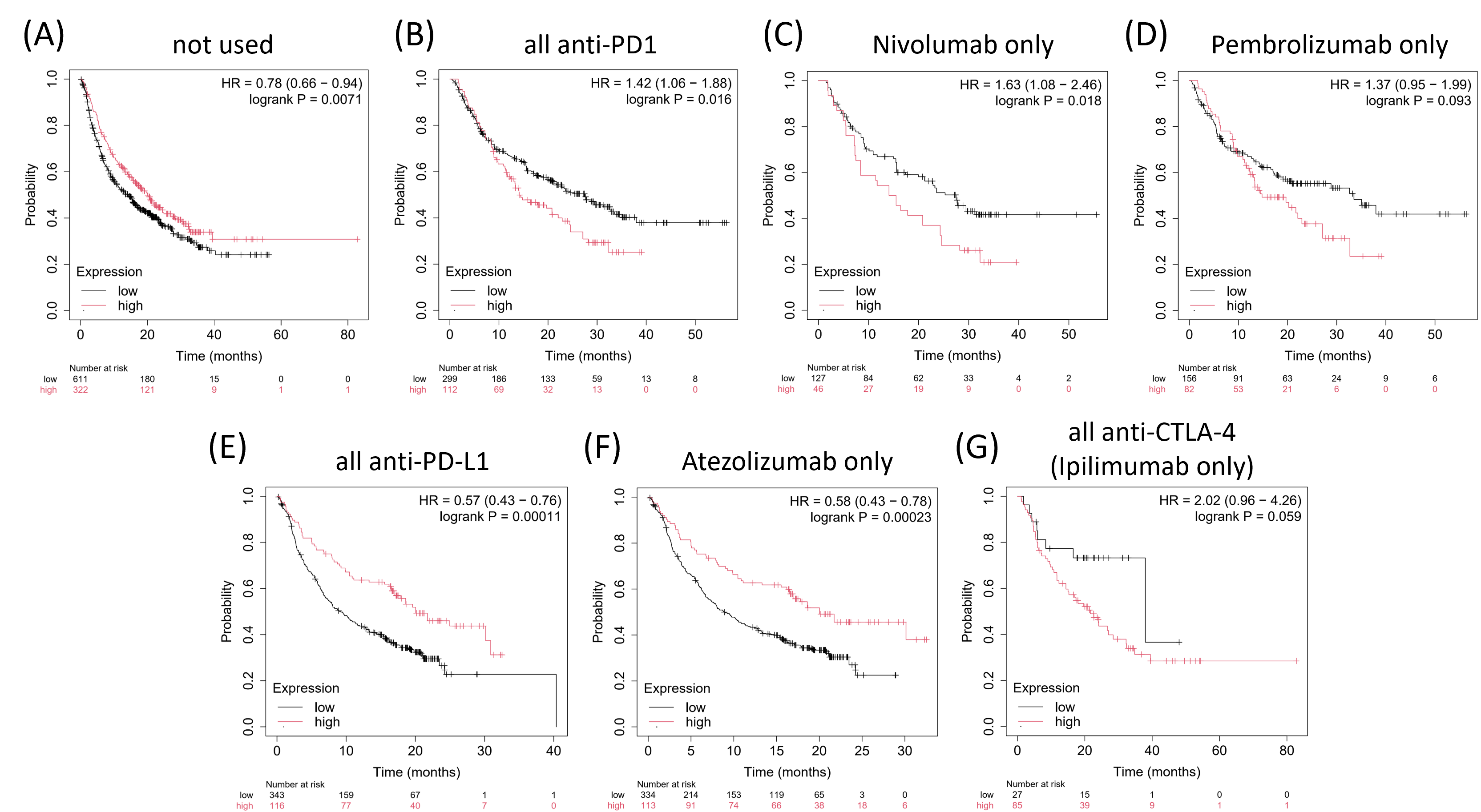

Supplement: Supplementary file 9 [file Image9.tif]

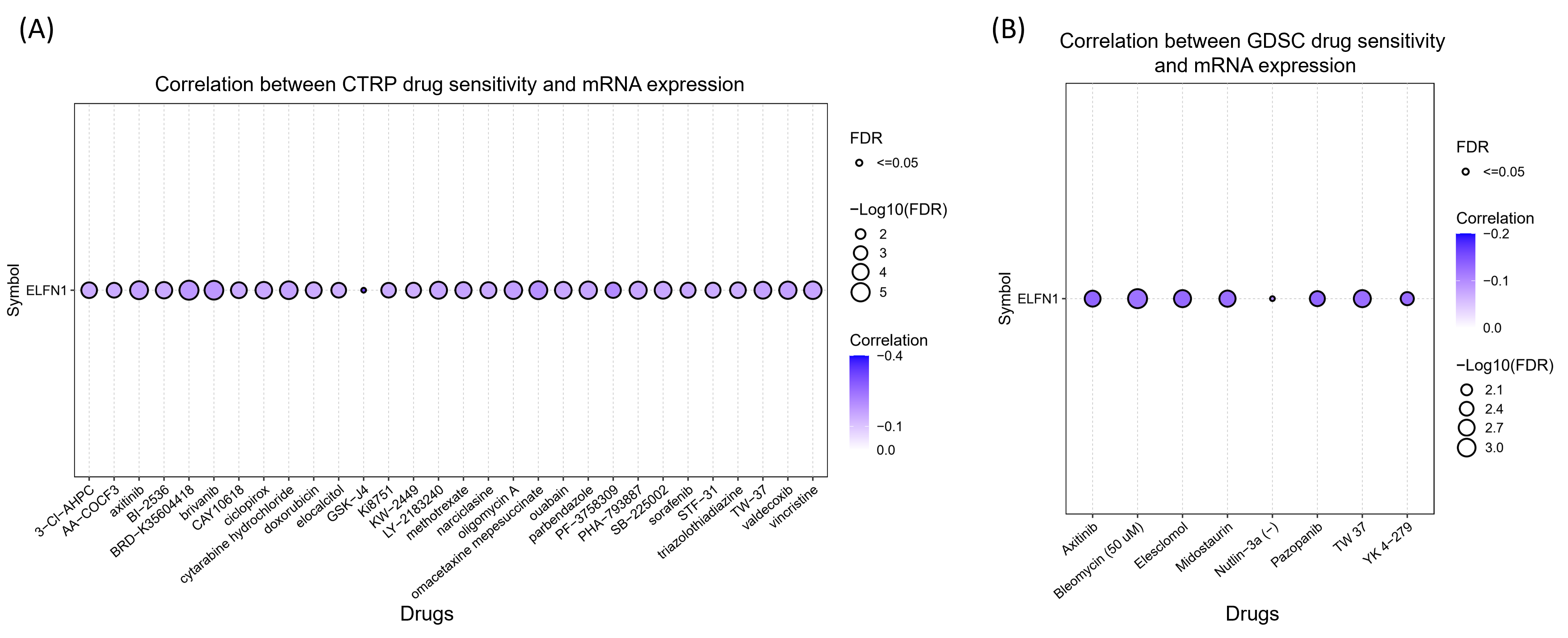

Supplement: Supplementary file 10 [file Image10.tif]
