# Supplementary material for: Pan-cancer analysis reveals ELFN1 as a novel prognostic biomarker and immunotherapeutic target associated with tumor microenvironment remodeling and promoting malignant phenotypes in colorectal cancer
Source: Front Oncol. 2025 Nov 20;15:1583277. doi: 10.3389/fonc.2025.1583277 (PMC12675275; doi:10.3389/fonc.2025.1583277)
Supplement: Supplementary file 11 [file Table1.docx]

Table S1. List of abbreviations.

| Abbreviations | Full name |
| --- | --- |
| ACC | adrenocortical carcinoma |
| BLCA | bladder urothelial carcinoma |
| BRCA | breast invasive carcinoma |
| CESC | cervical squamous cell carcinoma and endocervical adenocarcinoma |
| CHOL | cholangiocarcinoma |
| COAD | colon adenocarcinoma |
| DLBC | lymphoid neoplasm diffuse large B-cell lymphoma |
| ESCA | esophageal carcinoma |
| GBM | glioblastoma multiforme |
| HNSC | head and neck squamous cell carcinoma |
| KICH | kidney chromophobe |
| KIRC | kidney renal clear cell carcinoma |
| KIRP | kidney renal papillary cell carcinoma |
| LAML | acute myeloid leukemia |
| LGG | brain lower-grade glioma |
| LIHC | liver hepatocellular carcinoma |
| LUAD | lung adenocarcinoma |
| LUSC | lung squamous cell carcinoma |
| MESO | mesothelioma |
| OV | ovarian serous cystadenocarcinoma |
| PAAD | pancreatic adenocarcinoma |
| PCPG | pheochromocytoma and paraganglioma |
| PRAD | prostate adenocarcinoma |
| READ | rectum adenocarcinoma |
| SARC | sarcoma |
| SKCM | skin cutaneous melanoma |
| STAD | stomach adenocarcinoma |
| TGCT | testicular germ cell tumors |
| THCA | thyroid carcinoma |
| THYM | Thymoma |
| UCEC | uterine corpus endometrial carcinoma |
| UCS | uterine carcinosarcoma |
| UVM | uveal melanoma |
| ELFN1 | extracellular leucine rich repeat and fibronectin type III domain containing 1 |
| TME | tumor microenvironment |
| TIL | tumor-infiltrating lymphocyte |
| CAFs | cancer-associated fibroblasts |
| ICB | immune checkpoint blockade |
| TMB | tumor mutation burden |
| MSI | microsatellite instability |
| OS | overall survival |
| DSS | disease-specific survival |
| PFS | progression-free survival |
| DFI | disease-free interval |
| PFI | progression-free interval |
| CNV | copy number variation |
| SNV | single nucleotide variation |
| NEO | neoantigen load |
| HRD | homologous recombination deficiency |
| LOH | loss of heterozygosity |
| MMR | mismatch repair |
| HRR | homologous recombination repair |
| TIME | tumor immune microenvironment |
| PPI | protein-protein interaction |
