# Supplementary material for: Pan-cancer analysis reveals ELFN1 as a novel prognostic biomarker and immunotherapeutic target associated with tumor microenvironment remodeling and promoting malignant phenotypes in colorectal cancer
Source: Front Oncol. 2025 Nov 20;15:1583277. doi: 10.3389/fonc.2025.1583277 (PMC12675275; doi:10.3389/fonc.2025.1583277)
Supplement: Supplementary file 12 [file Table2.docx]

Table S2 The top 100 ELFN1 co-expressed genes identified on GEPIA2.0.

| Gene Symbol | Gene ID | PCC |
| --- | --- | --- |
| TMEM200C | ENSG00000206432.4 | 0.5 |
| AC110781.3 | ENSG00000176349.11 | 0.44 |
| TBC1D16 | ENSG00000167291.15 | 0.41 |
| SOX1 | ENSG00000182968.4 | 0.41 |
| HSPB2-C11orf52 | ENSG00000254445.1 | 0.4 |
| FAM53B | ENSG00000189319.13 | 0.4 |
| PNMA6A | ENSG00000235961.5 | 0.39 |
| LRRN4CL | ENSG00000177363.4 | 0.39 |
| CCDC140 | ENSG00000163081.2 | 0.37 |
| GAPDHS | ENSG00000105679.8 | 0.37 |
| RTTN | ENSG00000176225.12 | 0.37 |
| SLC6A17 | ENSG00000197106.6 | 0.37 |
| NAMA | ENSG00000271086.5 | 0.37 |
| EXOC3 | ENSG00000180104.15 | 0.37 |
| CTC-575N7.1 | ENSG00000251680.5 | 0.37 |
| PNMA3 | ENSG00000183837.9 | 0.36 |
| RGS12 | ENSG00000159788.18 | 0.36 |
| HSPB2 | ENSG00000170276.5 | 0.36 |
| ASB11 | ENSG00000165192.13 | 0.35 |
| HTR2B | ENSG00000135914.5 | 0.35 |
| PAX3 | ENSG00000135903.18 | 0.35 |
| LINC01531 | ENSG00000205786.8 | 0.34 |
| RP11-946P6.4 | ENSG00000279360.1 | 0.34 |
| CABLES1 | ENSG00000134508.12 | 0.33 |
| FLYWCH1 | ENSG00000059122.16 | 0.33 |
| RADIL | ENSG00000157927.16 | 0.33 |
| RP5-832C2.5 | ENSG00000215014.4 | 0.32 |
| LRRC39 | ENSG00000122477.12 | 0.32 |
| TSPAN4 | ENSG00000214063.10 | 0.31 |
| RASGRP3 | ENSG00000152689.17 | 0.31 |
| SH3RF3 | ENSG00000172985.10 | 0.31 |
| TYRP1 | ENSG00000107165.12 | 0.31 |
| CHAC1 | ENSG00000128965.11 | 0.31 |
| CTD-2380F24.1 | ENSG00000261195.1 | 0.31 |
| LGI3 | ENSG00000168481.8 | 0.31 |
| PQLC1 | ENSG00000122490.18 | 0.3 |
| TSPAN10 | ENSG00000182612.10 | 0.3 |
| PKNOX2 | ENSG00000165495.15 | 0.3 |
| MLANA | ENSG00000120215.9 | 0.3 |
| TEX41 | ENSG00000226674.8 | 0.3 |
| PTDSS2 | ENSG00000174915.11 | 0.3 |
| RP11-353N14.7 | ENSG00000275516.1 | 0.3 |
| ABR | ENSG00000159842.14 | 0.29 |
| GPR143 | ENSG00000101850.12 | 0.29 |
| C10orf11 | ENSG00000148655.14 | 0.29 |
| SH3RF3-AS1 | ENSG00000259863.1 | 0.29 |
| RGS20 | ENSG00000147509.13 | 0.29 |
| SERPINF1 | ENSG00000132386.10 | 0.28 |
| RP11-434D2.7 | ENSG00000266925.1 | 0.28 |
| HSD17B14 | ENSG00000087076.8 | 0.28 |
| LINC00403 | ENSG00000224243.1 | 0.28 |
| IGLL3P | ENSG00000206066.3 | 0.28 |
| TPGS1 | ENSG00000141933.9 | 0.28 |
| SLC7A8 | ENSG00000092068.18 | 0.27 |
| RENBP | ENSG00000102032.12 | 0.27 |
| ARMC9 | ENSG00000135931.17 | 0.27 |
| CA14 | ENSG00000118298.10 | 0.27 |
| SMO | ENSG00000128602.9 | 0.27 |
| CAPN3 | ENSG00000092529.22 | 0.27 |
| RAB3IL1 | ENSG00000167994.11 | 0.27 |
| OCA2 | ENSG00000104044.15 | 0.27 |
| RP11-273B19.2 | ENSG00000249727.1 | 0.27 |
| ILVBL | ENSG00000105135.15 | 0.27 |
| ST3GAL4 | ENSG00000110080.18 | 0.27 |
| LNP1 | ENSG00000206535.7 | 0.27 |
| SLC12A4 | ENSG00000124067.16 | 0.27 |
| CRTAC1 | ENSG00000095713.13 | 0.27 |
| TYRO3 | ENSG00000092445.11 | 0.26 |
| PIP4K2A | ENSG00000150867.13 | 0.26 |
| CITED1 | ENSG00000125931.10 | 0.26 |
| CTNS | ENSG00000040531.14 | 0.26 |
| CLCN7 | ENSG00000103249.17 | 0.26 |
| CIRBP | ENSG00000099622.13 | 0.26 |
| SCN4B | ENSG00000177098.8 | 0.26 |
| WDR81 | ENSG00000167716.18 | 0.26 |
| SOX6 | ENSG00000110693.15 | 0.26 |
| R3HCC1L | ENSG00000166024.13 | 0.26 |
| HDAC5 | ENSG00000108840.15 | 0.26 |
| SGCD | ENSG00000170624.13 | 0.26 |
| RP13-895J2.3 | ENSG00000256542.2 | 0.26 |
| SORBS3 | ENSG00000120896.13 | 0.25 |
| VAT1 | ENSG00000108828.15 | 0.25 |
| SLC7A4 | ENSG00000099960.12 | 0.25 |
| CTSF | ENSG00000174080.10 | 0.25 |
| RING1 | ENSG00000204227.4 | 0.25 |
| SCARF2 | ENSG00000244486.7 | 0.25 |
| RP3-462D8.2 | ENSG00000233577.6 | 0.25 |
| GYPC | ENSG00000136732.14 | 0.25 |
| CYGB | ENSG00000161544.9 | 0.25 |
| RP11-340F14.6 | ENSG00000274029.1 | 0.25 |
| LINC00404 | ENSG00000229520.1 | 0.25 |
| TECPR2 | ENSG00000196663.15 | 0.25 |
| ZNF517 | ENSG00000197363.9 | 0.25 |
| PDGFA | ENSG00000197461.13 | 0.25 |
| C6orf48 | ENSG00000204387.12 | 0.25 |
| SYDE1 | ENSG00000105137.12 | 0.25 |
| MAP2K2 | ENSG00000126934.13 | 0.25 |
| RAB38 | ENSG00000123892.11 | 0.24 |
| NKX2-4 | ENSG00000125816.4 | 0.24 |
| LHFPL3-AS1 | ENSG00000226869.6 | 0.24 |
