# Supplementary material for: Pan-cancer analysis reveals ELFN1 as a novel prognostic biomarker and immunotherapeutic target associated with tumor microenvironment remodeling and promoting malignant phenotypes in colorectal cancer
Source: Front Oncol. 2025 Nov 20;15:1583277. doi: 10.3389/fonc.2025.1583277 (PMC12675275; doi:10.3389/fonc.2025.1583277)
Supplement: Supplementary file 13 [file Table3.docx]

Table S3 GO pathways enriched by the top 100 ELFN1 co-expressed genes identified on GEPIA2.0.

| Ontology | Description | GeneRatio | BgRatio | pvalue | p.adjust | qvalue | Count | geneID |
| --- | --- | --- | --- | --- | --- | --- | --- | --- |
| CC | melanosome | 7/71 | 105/18675 | 1.44E-07 | 9.59E-06 | 8.64E-06 | 7 | TYRP1/MLANA/GPR143/SERPINF1/OCA2/CTNS/RAB38 |
| CC | Pigment granule | 7/71 | 105/18675 | 1.44E-07 | 9.59E-06 | 8.64E-06 | 7 | TYRP1/MLANA/GPR143/SERPINF1/OCA2/CTNS/RAB38 |
| CC | Melanosome membrane | 4/71 | 14/18675 | 1.87E-07 | 9.59E-06 | 8.64E-06 | 4 | TYRP1/GPR143/OCA2/RAB38 |
| CC | chitosome | 4/71 | 14/18675 | 1.87E-07 | 9.59E-06 | 8.64E-06 | 4 | TYRP1/GPR143/OCA2/RAB38 |
| CC | Pigment granule membrane | 4/71 | 14/18675 | 1.87E-07 | 9.59E-06 | 8.64E-06 | 4 | TYRP1/GPR143/OCA2/RAB38 |
| BP | Melanin biosynthetic process | 4/68 | 21/17910 | 1.08E-06 | 0.0009565 | 0.000836 | 4 | TYRP1/OCA2/CITED1/CTNS |
| BP | Melanin metabolic process | 4/68 | 22/17910 | 1.32E-06 | 0.0009565 | 0.000836 | 4 | TYRP1/OCA2/CITED1/CTNS |
| BP | Pigment biosynthetic process | 5/68 | 52/17910 | 1.54E-06 | 0.0009565 | 0.000836 | 5 | TYRP1/GPR143/OCA2/CITED1/CTNS |
| BP | Secondary metabolite biosynthetic process | 4/68 | 28/17910 | 3.63E-06 | 0.0016946 | 0.001481 | 4 | TYRP1/OCA2/CITED1/CTNS |
| BP | Pigment metabolic process | 5/68 | 66/17910 | 5.08E-06 | 0.0018941 | 0.001655 | 5 | TYRP1/GPR143/OCA2/CITED1/CTNS |
| MF | GTPase activator activity | 7/67 | 203/16967 | 1.50E-05 | 0.0029638 | 0.002504 | 7 | TBC1D16/RGS12/HTR2B/RASGRP3/ABR/RGS20/SYDE1 |
| MF | Enzyme regulator activity | 13/67 | 840/16967 | 2.07E-05 | 0.0029638 | 0.002504 | 13 | TBC1D16/RGS12/HSPB2/HTR2B/RASGRP3/ABR/RGS20/RENBP/CAPN3/WDR81/RING1/SYDE1/MAP2K2 |
