# Supplementary material for: Pan-cancer analysis reveals ELFN1 as a novel prognostic biomarker and immunotherapeutic target associated with tumor microenvironment remodeling and promoting malignant phenotypes in colorectal cancer
Source: Front Oncol. 2025 Nov 20;15:1583277. doi: 10.3389/fonc.2025.1583277 (PMC12675275; doi:10.3389/fonc.2025.1583277)
Supplement: Supplementary file 14 [file Table4.docx]

| FDA status | Drug name | PubChem CID | Binding energy (kcal/mol) | Response to drugs |
| --- | --- | --- | --- | --- |
| Clinical trial | ABT-737 | 11228183 | -10.0 | sensitivity |
| FDA approved | Cabozantinib | 25102847 | -8.9 | resistance |
| FDA approved | BLU-667 | 129073603 | -10.5 | resistance |
| FDA approved | JNJ-42756493 | 67462786 | -8.3 | resistance |
| Clinical trial | TAS-115 | 44247727 | -7.9 | resistance |
| Clinical trial | AZD-7762 | 11152667 | -10.2 | resistance |
| Clinical trial | CH-5183284 | 66555680 | -9.1 | resistance |
| Clinical trial | SCH-900776 | 46239015 | -7.1 | resistance |
| FDA approved | Norvir | 392622 | -8.4 | resistance |
| Clinical trial | PRN-1371 | 118295624 | -8.0 | resistance |
| Clinical trial | dimethylfasudil | 448043 | -8.3 | resistance |
| Clinical trial | Triapine | 9571836 | -8.9 | resistance |
| Clinical trial | Momelotinib | 25062766 | -9.1 | resistance |
| FDA approved | Bleomycin | 5360373 | -9.3 | resistance |
| FDA approved | Apatinib | 45139106 | -9.0 | resistance |
| Clinical trial | TAK-931 | 135564531 | -8.1 | resistance |
| FDA approved | Zoledronate | 68740 | -5.3 | resistance |
| Clinical trial | Quercetin | 5280343 | -8.5 | resistance |
| Clinical trial | PKM2 (9) | 772991 | -10.2 | resistance |
| Clinical trial | Telatinib | 9808844 | -9.1 | resistance |
| Clinical trial | OTS-964 | 89675898 | -8.9 | resistance |
| FDA approved | Tegafur | 5386 | -5.7 | resistance |
| FDA approved | Lenvatinib | 9823820 | -8.2 | resistance |
| Clinical trial | E-3810 | 25031915 | -9.3 | resistance |
| Clinical trial | Motesanib | 11667893 | -9.3 | resistance |
| FDA approved | Caffeic acid | 689043 | -6.4 | resistance |
| Clinical trial | benzaldehyde (BEN) | 240 | -5.2 | resistance |

Table S4. Molecular docking analyses.
